# Multidimensional Epistasis and the Transitory Advantage of Sex

**DOI:** 10.1371/journal.pcbi.1003836

**Published:** 2014-09-18

**Authors:** Stefan Nowak, Johannes Neidhart, Ivan G. Szendro, Joachim Krug

**Affiliations:** Institut für Theoretische Physik, Universität zu Köln, Cologne, Germany; ETH Zurich, Switzerland

## Abstract

Identifying and quantifying the benefits of sex and recombination is a long-standing problem in evolutionary theory. In particular, contradictory claims have been made about the existence of a benefit of recombination on high dimensional fitness landscapes in the presence of sign epistasis. Here we present a comparative numerical study of sexual and asexual evolutionary dynamics of haploids on tunably rugged model landscapes under strong selection, paying special attention to the temporal development of the evolutionary advantage of recombination and the link between population diversity and the rate of adaptation. We show that the adaptive advantage of recombination on static rugged landscapes is strictly transitory. At early times, an advantage of recombination arises through the possibility to combine individually occurring beneficial mutations, but this effect is reversed at longer times by the much more efficient trapping of recombining populations at local fitness peaks. These findings are explained by means of well-established results for a setup with only two loci. In accordance with the Red Queen hypothesis the transitory advantage can be prolonged indefinitely in fluctuating environments, and it is maximal when the environment fluctuates on the same time scale on which trapping at local optima typically occurs.

## Author Summary

Sexual reproduction is costly and often dangerous for the mating individuals, but nevertheless it is widespread in all higher organisms. To resolve this paradox, evolutionary theory searches for fundamental benefits in the recombination of genes that balance the cost of sex. Although many possible benefits have been proposed, they often rely on the assumption that each gene contributes independently to the fitness of an individual. Empirical studies indicate, however, that interactions between genes are prevalent in nature and strongly constrain the process of adaptation. Using a computational model, we show that these constraints lead to an advantage of recombination that is effective only in the early stages of adaptation to a new environment and turns into a disadvantage in the long run. A sustained benefit of recombination can arise if the environment is changing permanently, and we identify the conditions that maximize this benefit.

## Introduction

Sexual reproduction is a phenomenon that has existed for more than one billion years [1]. It is widespread in nature, which means that it must be advantageous compared to asexual reproduction, at least under certain conditions. Nevertheless, it is not well understood why this should be the case, since sex has several major disadvantages, e.g., the *two-fold cost of sex* [2], [3], which arises from the fact that two individuals are needed for reproduction without doubling the offspring in comparison to asexuals. But even if one considers mere genetic recombination, ignoring all the additional costs related to distinct sexes and mating, the advantage of genetic reshuffling is far from obvious [4]–[7]. In particular, useful structures in the genome may be disrupted by recombination giving rise to a *recombination load* [8].

In order to explain the prevalence of sexual reproduction, or at least recombination, many theories have been proposed. For example, *Muller's ratchet* [9], [10] and the related *deterministic mutation hypothesis* [11] explain the existence of sexual recombination through the difficulty of asexual reproduction to purge deleterious mutations. Other theories, like e.g., the *Red Queen hypothesis* [12], claim that the advantage of recombination may not lie in its ability to facilitate higher fitness values in the long run. Rather, it speeds up adaptation in order to be able to survive the competition with other co-evolving organisms and changing environments. Again, various mechanisms are conceivable that may cause such a speedup of adaptation. Fisher [13] and Muller [14] first described what is now known as the *Fisher-Muller effect* (which is closely related to the *Hill-Robertson effect* [10], [15]): if in a population two beneficial mutations occur, recombination of the two genomes can lead to offspring harboring both mutations, while in asexual populations the double mutant can be created only if one of the mutations reoccurs by chance on the background of the other mutant. The *Weismann effect*, on the other hand, ascribes the advantage of sex to the creation of variation amongst siblings [16]. The advantage of the Weismann effect is implied in *Fisher's fundamental theorem*, which states that the mean fitness gain of a population under natural selection is proportional to its additive genetic variance [13].

To be able to assess the effectiveness of the proposed mechanisms, it is necessary to make some simplifying assumptions. In order to describe evolutionary processes and adaptation, Wright introduced the notion of a *fitness landscape* [17]. Fitness is a real number that is a measure for the reproductive success of an organism, and the fitness landscape is a mapping that assigns fitness values to genotypes [18]. Throughout the paper the term fitness landscape refers to this *genotype landscape*, to be distinguished from the mean fitness landscape, also known as the adaptive landscape, which describes the mean fitness of a population as a function of its genotype or allele frequencies [19], [20]. Each site of the landscape corresponds to a possible genotype and the individuals are distributed among these sites. While mutation and recombination enable the exploration of formerly unpopulated sites, selection leads to the concentration on genotypes with particularly high fitness. Thus, the population as a whole performs an uphill walk through the landscape and hence, on average, an increase of fitness in time can be expected.

In recent works, the importance of *epistasis* for the evolutionary dynamics has been highlighted: If the change of fitness associated with a mutation depends not only on the mutation itself, but also on the configuration of the rest of the genome, the fitness landscape is called *epistatic* [21]. If the fitness effect of a mutation is altered by the state of the other loci only in magnitude but not in sign, the effect is called *magnitude epistasis*. If a mutation can be beneficial or deleterious depending on the rest of the genome, the effect is called *sign epistasis* [22]. Epistasis affects the mutational accessibility [23]–[27] of fitness landscapes as well as the number of fitness optima, i.e., genotypes that have higher fitness than all of their single-mutant neighbors [28]–[30], and the length of adaptive walks leading to such states [31]–[37]. While the absence of sign epistasis implies the absence of multiple fitness optima, yielding so called *smooth* landscapes, sign epistasis leads to *rugged* landscapes, which may contain many local fitness optima.

For a long time, the absence of experimental data lead to an exclusively theoretical approach to the study of fitness landscapes. Over the last years an increasing number of experimental studies devoted to the analysis of empirical fitness landscapes and a comparison to theoretical models have been reported [18], [23], [24], [38]–[41]. Many of these empirical data sets suggest that realistic fitness landscape are not smooth but show a varying amount epistasis. Therefore, some of the effects described above, which assume that mutations accumulate additively (e.g., the Fisher-Muller effect), may become less important for the advantage of recombination, even if they are crucial on smooth landscapes. On the other hand, on a rugged landscape it becomes more important for a population to be able to escape from local fitness optima and not only to find genotypes with larger fitness as fast as possible. It was shown in [42]–[44] that the time a population takes to cross a fitness valley may have a minimum at a small (but non-zero) recombination rate. However, when the recombination rate is increased further, the escape time increases rapidly and even diverges in the limit of infinite population size [45], [46]. Hence the mechanisms conveying a benefit of recombination are in competition with the disadvantageous effect of trapping at local optima.

So far, only a few studies have addressed the problem of recombination on large fitness landscapes containing sign epistasis [47]–[52] and the criteria that determine whether recombination is of advantage on some specific landscape remain unclear. Of particular relevance to the present work are the results of a recent study on recombination in an epistatic fitness model in which two regimes of evolutionary dynamics were identified [53], [54]: One where recombination is strong compared to selection, linkage is weak and selection acts mainly on the allele frequencies, and a weak recombination regime, where linkage is strong and the dynamics leads to the condensation of the population around particularly fit haplotypes. In the following, we study the (dis)advantage of recombining populations compared to non-recombining ones on multidimensional, epistatic fitness landscapes with tunable ruggedness and strong selection, resulting in strong linkage. Since this corresponds to the weak recombination regime of [53], [54], a parametrization in terms of linkage disequilibria and allele frequencies is not suitable and a genotype-based description is used.

We find that whether recombination is of advantage or not depends crucially on the time at which the advantage is evaluated. Our results are based on simulations of sexual and asexual Fisher-Wright dynamics on landscapes generated according to the Rough Mount-Fuji model [24], [55]–[57] (see **Models**). In this model the ruggedness of the landscape can be easily tuned between the additive and fully random limits, and it has been shown to faithfully reproduce many statistical features of real fitness landscapes [24], [39], [40] We find numerically that in most cases recombination is advantageous at intermediate timescales. However, this advantage is only transitory and non-recombining populations always overtake the recombining ones in the evolutionary race at long times. The following sections focus on describing and explaining the temporal patterns of the relative fitness evolution in static landscapes. We then consider evolutionary dynamics on fitness seascapes [58], i.e. time dependent fitness landscapes, to model a population in a changing environment, and show that under this scenario the advantage of recombination becomes stationary in agreement with the Red Queen hypothesis. Although a transitory advantage of recombination has been found and explained previously for different fitness models (see for example [4]), we will argue that the ruggedness of the landscape introduces a very different origin of this phenomenon. The same holds true for the associated maintenance of the advantage on time-dependent landscapes (see [7], [20] and references therein).

## Results

### General phenomenology

In this article, we are interested in the advantage recombination provides to adaptation compared to dynamics consisting only of mutation and selection. Our model aims at describing haploid populations of facultative sexuals, which are common among microorganisms such as bacteria, viruses or rotifers. For this purpose we introduce the parameter 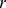 as the fraction of the population that recombines. As recombination requires the coexistence of multiple mutant clones to have an effect, in the following, we choose the population size 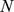 and genome-wide mutation probability 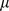 such that 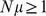, which implies that several mutations occur each generation (see **Models** for a precise definition of the model parameters). In order to quantify the advantage of recombination, we consider the difference of mean fitness of the two processes, 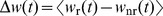, where 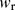 and 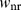 denote the population mean fitness at time 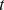 of a recombining (recombination fraction 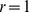) and a non-recombining (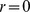) population on the same realization of the fitness landscape, respectively. Angular brackets 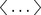 denote averaging over (typically a few thousand) runs of the dynamics. Each simulation starts with the whole population placed at the reference genotype 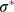 of a new landscape, created according to the RMF model with 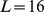 biallelic loci (see **Models**). Since the additive fitness component in the RMF model increases linearly with distance from 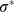, this corresponds to an initial condition of low fitness mimicking a poorly adapted organism, e.g. after a change of environment, which is chosen here in order to provide a long period of adaptation. Note, however, that the overall phenomenology remains valid for different choices of the starting genotype.

As an alternative measure we considered the probability that the recombining population has a higher mean fitness than the non-recombining one, 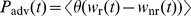, where 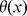 denotes the Heaviside theta function that equals unity for 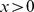 and zero for 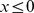. Another possibility would be to consider a single population into which a modifier allele is introduced that determines whether an individual proliferates sexually or asexually [59]. In this setup recombining and non-recombining populations are under direct competition. The fraction of sexuals in a population could then again be used as an indicator for the (dis)advantage of recombination. As can be verified in fig. S1, both alternatives behave similarly to 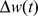. The semblance to 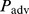 implies that 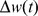 is not dominated by a few exceptional runs of the dynamics. Hence it is an appropriate choice for an indicator to assess whether recombination is typically of advantage or not and we will mainly concentrate on this measure throughout the article.

**Figure 1:**
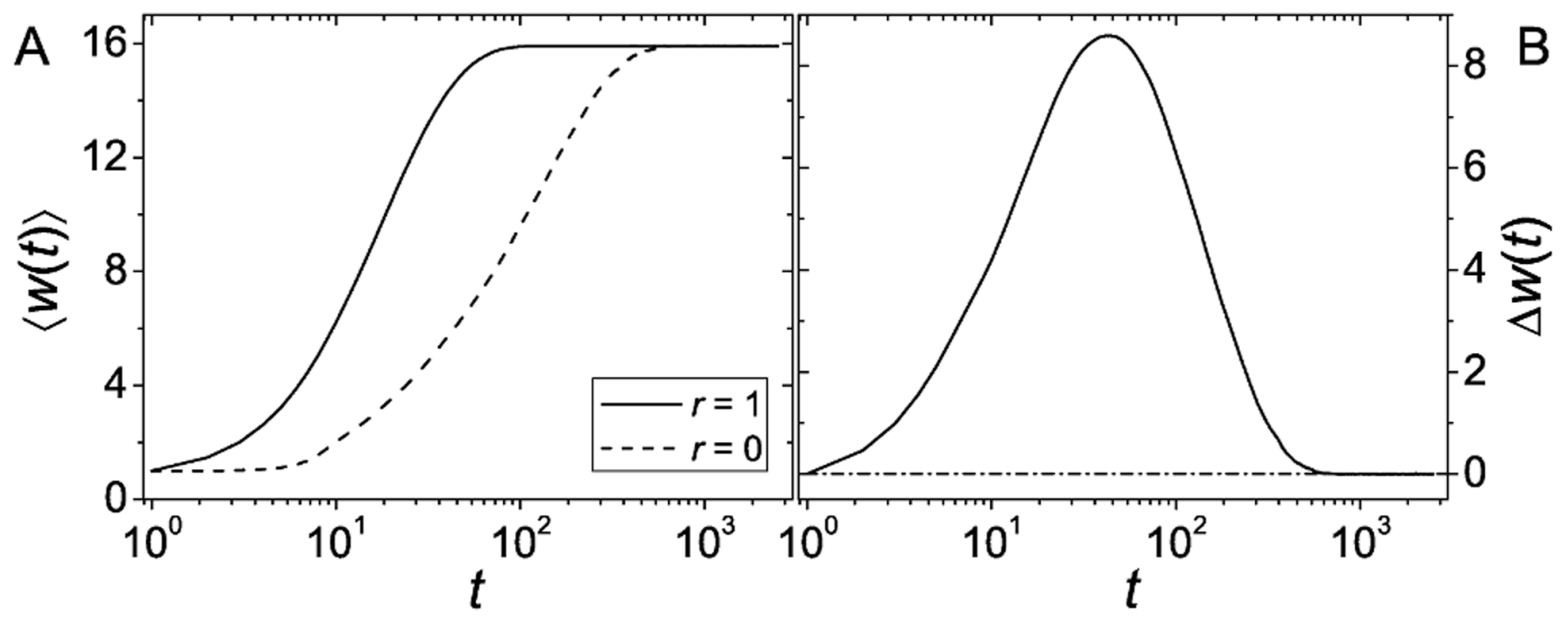
Advantage of recombination on a smooth fitness landscape. The figure shows time series of (A) mean fitness 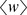 and (B) difference of the mean fitnesses 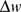 for recombining (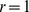) and non-recombining (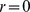) populations in an additive landscape. Time 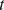 is measured in discrete generations. Population parameters are 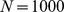, 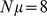, and the slope of the additive fitness landscape is 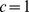. Although not visible to the naked eye, 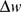 converges to a value below zero at long times.

Before considering fitness landscapes with sign epistasis, it is instructive to have a first short look at purely additive landscapes. As can be seen in fig. 1B, 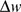 increases in time until it reaches a maximum and subsequently declines to a value around, actually slightly below, zero. This behavior is easily understood when considering the individual curves for 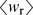 and 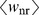 (see fig. 1A). For small times 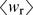 increases more rapidly than 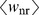 until it converges to a maximal level. Subsequently 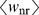 catches up and converges to a slightly higher value (see e.g. [60] for an analytical approach to a similar setting in the weak selection limit). It is easy to check that the initial increase of 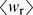 and 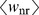 stops when the populations reach the global optimum of the landscape. This setup without epistasis has been studied in some detail in [61], where the initial advantage of recombination was attributed to the Fisher-Muller effect. The slight disadvantage of recombination at long times reflects the recombination load, i.e., the fact that recombining populations maintain a larger amount of genotypic diversity, which means a larger number of individuals carrying not the fittest but neighboring genotypes. In the setup with direct competition [59], the non-recombining subpopulation would have a selective advantage at this point which decreases the amount of recombination. This is known as the reduction principle [62]. Note however that if selection is weak enough, recombining populations will asymptotically achieve higher mean fitness values than non recombining ones due to the Hill-Robertson effect [63].

**Figure 2:**
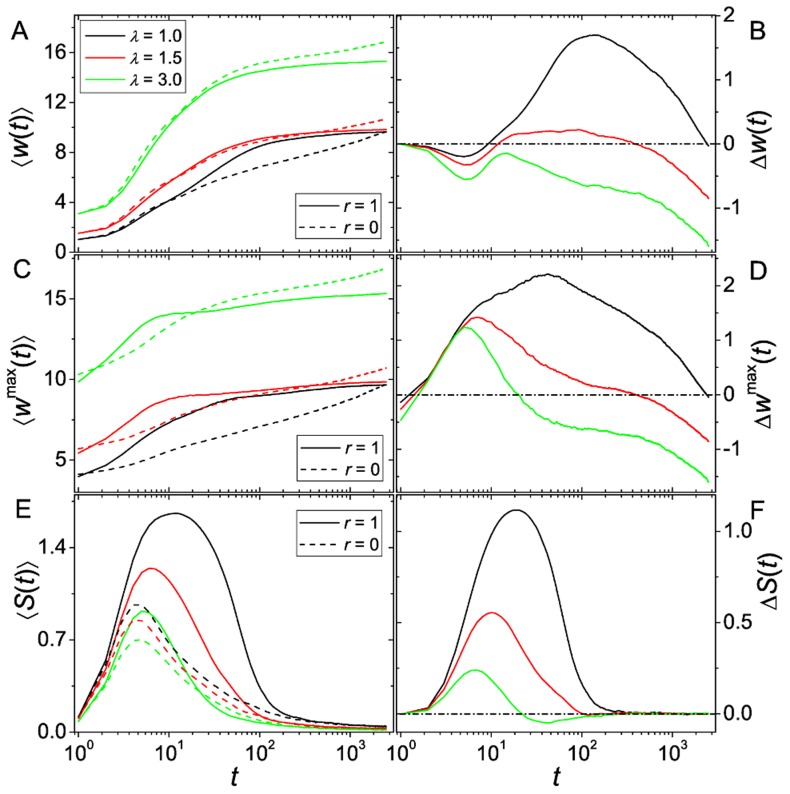
Dependence of the recombinational advantage on fitness landscape ruggedness. The figure shows time series of different observables for varying ruggedness parameter 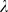. (A, B) Mean fitness 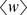. (C, D) Fitness of the fittest individual 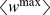. (E, F) Entropy 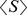 of the genotype frequency distribution as a measure for genetic diversity. Left columns shows quantities for populations with 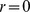 and 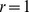 separately, the right column shows the difference. Parameters are 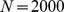, 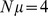, and 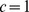.

**Figure 3:**
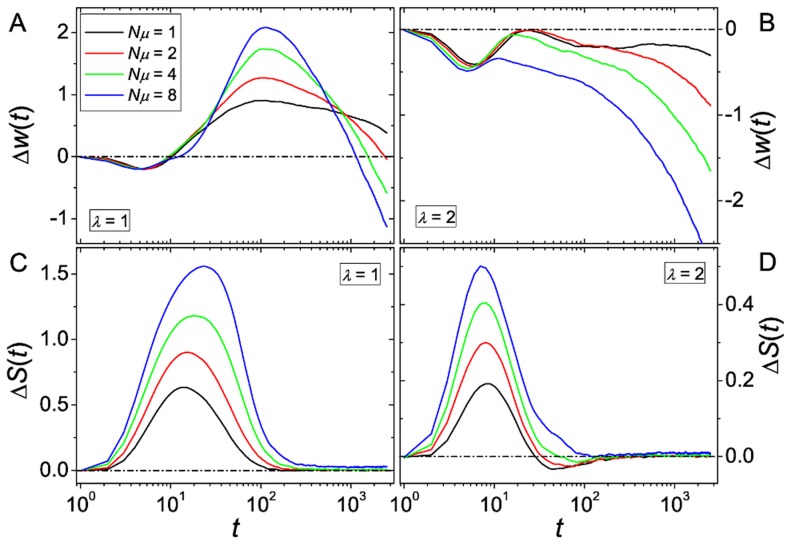
Dependence of the recombinational advantage on mutation supply. The figure shows time series of mean fitness difference 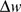 (A, B) and entropy difference 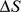 (C, D) for varying mutation rate 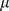 and two different values of the ruggedness parameter 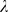. The population size is 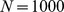 and 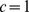.

Let us now turn to rugged landscapes. In fig. 2B we show results for 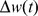 corresponding to three different choices of 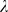, the parameter of the exponential random variables describing the random component of the RMF fitness landscape (see **Models**); further examples are shown in figs. 3A, B and S4A, B. Now 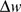 becomes clearly negative at long enough times, and not (close to) zero as before. This means that non-recombining populations always overtake recombining ones. The origin of this long term disadvantage can again be better understood by considering 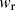 and 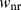 separately (see fig. 2A). A crucial observation is that the increase of fitness of recombining populations levels off very fast at some point, while for non-recombining populations it increases slowly but more steadily. Unlike in the case without epistasis, the leveling off for recombining populations is not because the global optimum has been reached, and a higher genetic variance induces a recombination load. As we will show later on, the explanation is that populations are trapped for extremely long times at local maxima. We will argue that such trappings are the more likely, and the trapping times the longer, the larger the recombination rate. Therefore, in rugged landscapes, large recombination rates are always disadvantageous at long times. Nonetheless, figs. 2B, 3A, B and S4A, B also clearly show a transitory regime where recombination can be advantageous, depending on the parameters controlling the ruggedness of the landscape and the dynamics.

The temporal pattern observed for 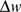 appears to be qualitatively the same on any rugged landscape, including models other than RMF (such as the NK-model [64], see fig. S3). At very short times non-recombining populations are more efficient in adapting, causing an initial decrease of the 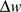. Shortly after 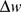 increases again, showing that now recombination is more efficient. Finally, this period of fast fitness increase of the recombining population comes to an end and the non-recombining populations again display a faster fitness increase. Whether the intermediate period of increased effectiveness of recombining populations is long enough to catch up and temporarily overtake the non-recombining populations depends on the systems parameters.

Albeit of transitory nature, this advantage of recombination at intermediate timescales may be essential for a population that is transferred to an environment where it is initially poorly adapted. Although the effect is transient, the additional gain in adaptive effectiveness could determine if the population adapts in time before being displaced by other organisms. Further, if the period of positive 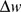 is long enough, a non-recombining populations might be displaced by the recombining variant before it can make use of its long time advantage. The remainder of this article is devoted to explaining the processes responsible for the observed temporal pattern of 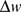.

### Short time advantage of non-recombining populations

As can be verified in figs. 2B, 3A, B there is a short period at small times where 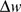 decreases, implying an advantage of non-recombining populations (see also S4A, B in the supplementary material). We will argue that the initial decrease of 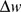 is a consequence of the monomorphic initial condition, or, more precisely, of the property of recombination to create larger diversity which is also at the heart of the concept of recombination load. Non-recombining populations rapidly select some fit neighboring state and reside there almost monomorphically until a new fitter mutant is created. The recombining populations also mostly occupy such fit neighbors, but in addition contain a considerable fraction of other genotypes that are often less fit. This lowers the average fitness but is not necessarily a disadvantage for further adaptation, as the diversity can augment the evolvability of the population.

To show that recombining populations do indeed create a larger diversity, we have calculated the Shannon entropy 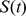 of the population distribution, which is defined as 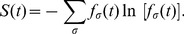
(1)


Here the sum runs over all genotypes, and 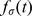 is the fraction of the population with genotype 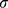 at time 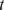. The more homogeneously the population is distributed among the different states, the larger is the value of 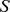, while for a monomorphic population 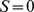. In the following we consider 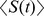, where the brackets again denote averaging over ensembles of simulation runs, each in a different realization of the landscape. An alternative measure for the diversity is the additive genetic variance 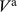, which is the variance of the part of the fitness which is inherited. In our setup, this is just the variance of the distance to the reference sequence in the population, 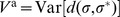 (see **Models** for the definitions of 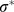 and 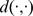). As can be seen in fig. S2, 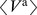 and 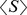 behave very similarly and we will therefore, in the following, only discuss the entropy.

For recombining as well as non-recombining populations 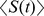 initially increases at short times but then decreases again to a very low level (cf. fig. 2E). In the initial period 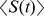 is always larger for recombining than for non-recombining populations. Further, the initial increase of 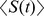 is faster and also more prolonged. This clearly shows the ability of recombination to build up a larger amount of diversity, supporting our claim that the initial decrease of 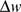 is due to recombining populations distributing among a large number of states many of which are probably not very fit.

However, there are at least two other mechanisms that could contribute to the decrease: First, non-recombining populations might be more efficient in finding particularly fit mutants, and second, non-recombining populations might be more effective in selecting fit mutants once they have been created.

The first claim is easy to disprove. In fig. 2D we plot 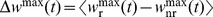, where 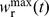 and 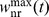 denote the fitness of the fittest observed mutants at time 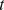 in recombining and non-recombining populations, respectively. The temporal pattern of 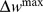 is similar to the one of 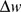, but there is no dip at short times (except perhaps a very small disadvantage at the first two time steps). This shows that even at short times recombining populations are better in finding particularly fit mutants than non-recombining ones. Note also that these particularly fit mutants have a high probability to take over the population due to selection and therefore determine the future evolution. This might be the reason why the temporal patterns of 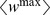
's and 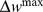 are similar in shape to the ones of the 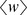
's and 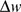, but with all features shifted to shorter times (see figs. 2A, B, C, D). In fact, in order to evaluate which dynamics performs better, it might be more appropriate to consider 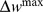 instead of 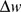.

The second claim, that the initial decline may reflect non-recombining populations being more effective in selecting fit mutants, is not that easy to reject. In fact, the very small negative values of 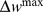 at the first two time steps might be due to this reason. In our setup recombination acts as an additional source of genetic drift, which augments the probability of extinction of newly found beneficial mutants. As a consequence beneficial mutants fix less effectively. However, for large populations this effect should be small.

### Period of advantage of recombination

A large part of the literature about sexual reproduction deals with the question by which mechanisms recombination can speed up evolution, two of the most prominent ones being the Fisher-Muller and the Weismann effect. Despite the considerable amount of dedicated research, it has proven rather difficult to assess the impact of the different mechanisms, especially when considering rugged fitness landscapes for large genomes. In this article, we are mainly interested in understanding why these mechanisms seem to fail at long times. Therefore, we restrict ourselves to the observation that the standard mechanisms can be efficient at intermediate times, as shown by the increase of 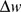. This regime depends in rather complex ways on the parameters controlling the landscape ruggedness and the dynamics.

The Fisher-Muller effect consists in the advantage gained by combining mutations present in the population to obtain new, fitter recombinants, allowing the genotype space to be explored in large jumps. This mechanism should be efficient on a RMF landscape as on such a landscape the fitness increases on average the more mutations are accumulated with respect to the initial genotype 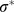. Of course, recombination can also lead to the loss of mutations. But as the mutants containing many mutations are expected to be particularly fit, they have a large probability to take over the population due to selection, whereas less fit recombinants are rapidly purged and do not affect the future development of the population.

In the presence of epistasis, one could naively expect an even larger advantage of recombination, because the neighboring states of highly populated genotypes are typically of low fitness, whereas the large jumps due to recombination may lead to states much further uphill the landscape that have a high probability to be more fit. As we will see later, this intuition is largely wrong, since recombination enhances the trapping at local maxima. Nonetheless, before the population falls into such a local maximum, this mechanism may still be efficient.

#### Effect of landscape ruggedness

In fig. 2B we plot 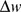 for three different choices of the ruggedness parameter 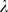. While the advantage of recombination in the intermediate regime decreases monotonically with increasing ruggedness, the effect of 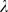 on the individual mean fitnesses 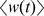 is less clear. Figure 2A seems to suggest that both 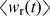 and 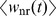 increase with increasing 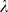, but this behavior is confounded by the fact that the random component of the landscape has mean 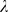, and therefore the mean fitness level shifts with increasing 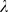 (see **Models**). There are also cases where higher mean fitness values are reached for smaller 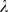, but it remains true that 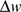 decreases with increasing 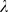.

A possible explanation for the decrease of the adaptive advantage of recombining populations with increasing landscape ruggedness lies in the link to population diversity, which is a prerequisite for both the Fisher-Muller effect and the Weismann effect. In rugged landscapes diversity is suppressed, because selection focuses the population onto high-lying fitness peaks and ridges separated by valleys of much lower fitness. On smoother landscapes, on the other hand, many mutants have comparable fitness values and will therefore coexist for longer times. This effect can be observed in recombining as well as in non-recombining populations, as illustrated by the behavior of the corresponding mean entropies fig. 2E. Recombining populations create a larger diversity than non-recombining ones, such that the entropy difference 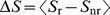 is positive at small times and increases until it starts to decrease at some time that shortly anticipates the decrease of 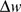 (compare figs. 2B and F). Importantly, fig. 2F also shows that 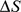 decreases with increasing 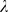.

#### Effect of mutation supply and population size

A simple relation between population diversity and rate of adaptation is suggested by Fisher's fundamental theorem, which states that the mean fitness increase is proportional to the fitness variance [13]. In the following we show that this argument is not generally valid on rugged fitness landscapes. For this purpose we manipulate the population diversity through the mutation supply rate 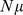, the number of mutants created in each generation. With increasing 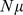 the population diversity quantified by the entropy 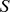 increases for recombining as well as for non-recombining populations (not shown). The effect is again larger for recombining populations, as can be verified in figs. 3C, D. However, whereas for the smoother landscape (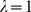) the increase in diversity is accompanied by an increase in 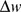, the opposite is the case for the more rugged landscape (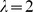) (figs. 3A and B).

The reason for this negative influence of increased diversity on the advantage of recombination on very rugged fitness landscapes may be the following: The large diversity at initial times causes the coexistence of many different mutants, but only the most fit mutants have the chance to get selected. On very rugged landscapes, such mutants have a relatively high probability to be local optima. As will be shown in the next section, once almost the entire population resides on such a local optimum, the recombining population essentially stops adapting. On less rugged landscapes, on the other hand, the probability to find a local optimum is smaller and hence the population can derive an extended benefit from the larger diversity.

A similar but less pronounced change in population diversity also mediates the effect of the population size on the advantage of recombination, when 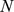 is varied at fixed mutation supply rate 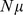. As 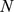 is increased, the strength of selection is enhanced while genetic drift decreases. In addition, we claim that the diversifying effect of mutation is reduced when 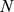 increases, leading to a decrease of the entropy 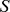. In the presence of recombination, this decrease is further enhanced, leading to a decrease of 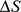 (see figs. S4C, D in the supplementary material). This behavior can be understood from the following argument: In each time step, 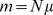 mutants are created on average. The frequencies 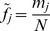 corresponding to the different genotypes of newly produced mutants are inversely proportional to 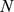. Therefore, the larger 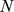, the smaller the fraction of the population exploring new genotypes. This, in turn, leads to a lower diversity for both recombining and non-recombining populations. But as the frequencies of mutants decrease, so does the frequency of new individuals generated by recombination. Given two new states populated with frequencies 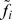 and 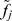, on average, a fraction 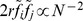 of the population will be provided by the recombination of their genotypes. Thus, for increasing 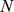, the absolute number of offspring of such pairings is diminished, suppressing the exploration of additional unpopulated sequences by recombination. This causes the decrease of 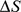. Just as for the case where 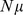 was varied (fig. 3), the decrease in diversity leads to a somewhat smaller adaptive advantage of recombination in relatively smooth landscapes, while the opposite is true in more rugged landscapes (see figs. S4A, B in the supplementary material).

#### Infinite populations

A question that naturally arises here is whether the transient advantage of recombination still exists in the limit 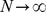. A priori this is not at all obvious. If the only advantage of recombination would lie in the exploration of formerly undiscovered genotypes, it would be irrelevant in the infinite 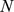 limit, as in this limit all genotypes are populated after the first mutational step. On the other hand it is also not clear if trapping at local fitness maxima will still occur if all, even distant, maxima are populated.

In fact, trapping has been observed in the infinite population dynamics on a specific, rugged empirical fitness landscape [49]. To clarify whether this is a common phenomenon, we performed simulations in the infinite 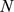 limit on RMF landscapes. The algorithm allows for an infinite number of multiple mutations in a single time step and is described in **Models**.

**Figure 4:**
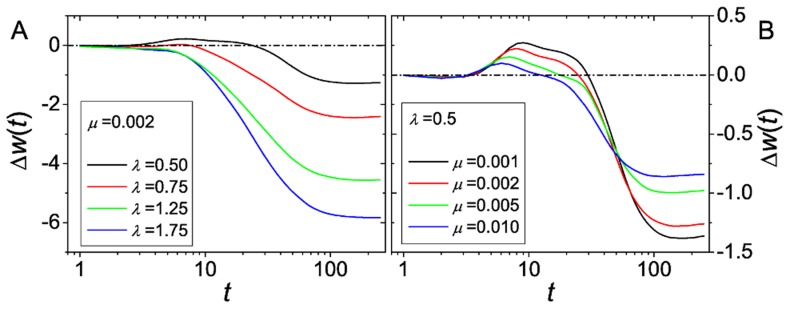
Advantage of recombination for infinite populations. The figures show the results of simulations in the infinite population size limit. (A) shows a comparison for different choices of 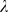 and (B) a comparison for different mutation rates 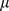.

Figure 4A shows the results of simulations with 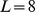 and various choices of the landscapes ruggedness. The general shape of 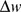 is similar to that observed in finite populations. At least for less rugged landscapes, recombination is still beneficial on intermediate time scales. Hence we conclude that recombination not only provides an advantage by exploring a larger fraction of the landscape but may also speed up the adaptation process. With increasing ruggedness, trapping events happen more often and earlier, eventually eliminating the transient advantage of recombination.

A comparison of different mutation rates is shown in fig. 4B. Larger values of 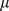 lead to a faster fitness increase for mutation-only processes, decreasing the advantage of recombination at intermediate times. On the other hand, the mean fitness reached by the non-recombining populations in the long time limit (where 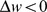) is higher for small values of 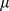. This is due to the mutational load: the larger mutation rate leads to a larger fraction of the population spreading over the landscape, even after the global maximum has already fixed.

### Long time disadvantage of recombination

Although the mechanisms leading to an advantage of recombination work efficiently at intermediate times, we have already seen that this advantage is only transitory. In the following, this finding will be explained on the basis of known results concerning the dynamics of evolution with recombination in a two locus system. For that purpose we recall some of these results that will prove essential to understanding the dynamics in high-dimensional fitness landscapes, see [42]–[46], [65] for details.

Consider a population that reached a local fitness maximum. To proceed and reach states with higher fitness, a fitness valley has to be crossed. Populations that do not recombine can do so in two ways. The first way consists in first fixing the population at a valley genotype and subsequently fixing it at some state fitter than the initial state. Here, “fixation” is meant in an approximative way, in the sense that the population becomes strongly concentrated on some genotype. The second way to cross the valley is to produce only a small population of mutants in the valley, which can then further mutate, giving rise to new mutants at a larger distance from the initial genotype. These initially few multiple mutants can, if they are fit enough, take over the population. Both modes of valley crossing are strongly suppressed with increasing population size 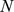, but for large enough 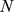 the second one dominates [42], [65].

Recombination opens up a third path. Mutants can be produced at various states in the valley and these mutants can then recombine to produce fitter mutants at larger distance from the initial genotype. But apart from providing a new path to escape from a local maximum, recombination also yields a mechanism that can make the escape even more difficult. Suppose that a few mutants have been produced on a state with larger fitness than the initial one. Since nearly the whole population is still located on the initial (peak) state, recombination replaces these fit mutants by unfit valley genotypes with high probability, and selection will then resort them to the initial point.

Whether recombination is of advantage or disadvantage for such escapes depends strongly on the selective benefit of the target state and the selective disadvantage of the valley states in relation to the fraction of recombining individuals 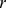. Most noteworthy, if 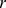 is larger than some critical value *r* ^*^ which depends on the selection coefficients, the escape time in the two-locus landscape increases exponentially with 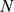, at 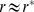 as 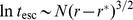 [44]. In contrast, the escape times for non-recombining populations increase only algebraically in 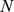 [42], [65]. In our multidimensional landscape model, each possible escape path from a local peak involves different fitnesses and hence a different value of 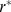. For large 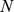 it follows that the escape times will become extremely large compared to those of non-recombining populations, if a major part of the population reaches a local maximum at which all the 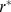
's are smaller than 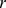. The recombining populations are trapped. As a consequence, the advantage that the recombining populations could build up by means of the Fisher-Muller and the Weismann effect is lost, while the non-recombining ones can slowly catch up and overtake. In the limit of infinite population size these local maxima in the genotypic fitness landscape can induce multiple equilibrium states, in the sense that, depending on the initial condition, the population remains centered around a suboptimal fitness peak for all times. Again, this phenomenon occurs when the recombination rate exceeds a certain threshold and is absent without recombination [45], [46], [66], [67].

To show that the trapping scenario known from the two locus system is also effective in high-dimensional landscapes, we have measured the fraction of escape events over the number of trapping events, 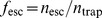, observed up to run time 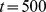 as a function of the recombination rate 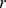. Note that, because each escape is preceded by a trapping event, 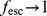 for very long times. However, in the cases studied here, escapes are very rare, and 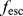 is still a useful measure. In the simulations we considered a population as trapped if 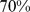 of the individuals share the genome corresponding to the local maximum and an escape event is registered if, for a population that was marked as trapped, the fraction of mutants on the local maximum falls below 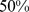.

**Figure 5:**
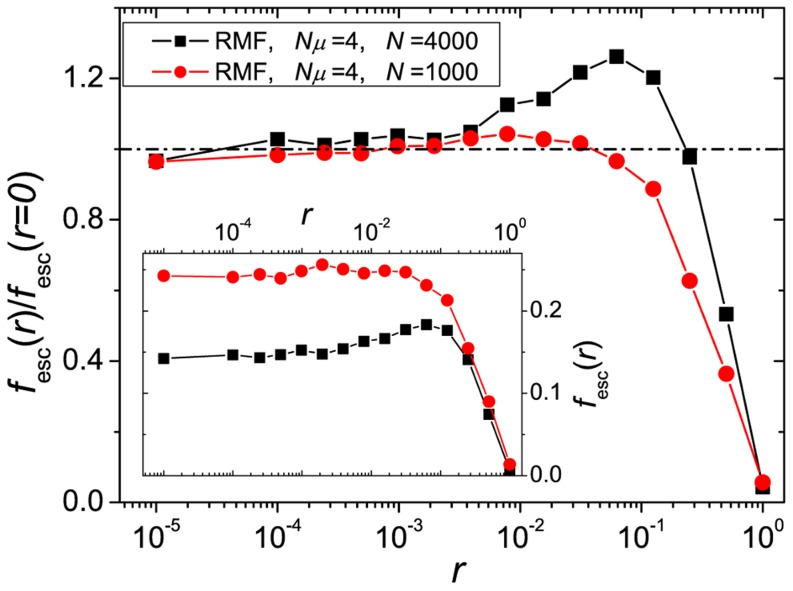
Escape from local maxima. Fraction of escape events 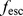 on RMF landscapes with 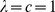 in dependence on the recombination rate 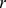 and normalized to the value at 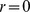. The inset shows the values without normalization. For suitable choices of the population parameters 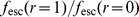 has a maximum.

As expected, the number of escapes drops rapidly when 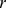 approaches unity (fig. 5). As 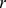 decreases, strong trapping, that only occurs for local maxima for which all the 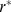
's are smaller than 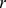, gets less likely and the number of escapes converges to that expected in the absence of recombination. The decrease of 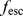 when increasing 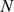 (see inset of fig. 5) is not surprising since both the escape under the influence of only mutation, as well the escape in the presence of recombination, are suppressed when increasing 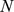 [42], [43], [65]. Interestingly, for sufficiently large 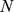, there exists an intermediate regime of 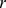 values, where escapes are more frequent with, than without recombination. This can again be explained by the results of the two locus model. There, it was shown that, if the number of individuals at the valley states is dominated by fluctuations, the escape time has a minimum [43], [44]. We claim that this minimum in the escape times is the cause of the maximum in the escape fraction, which is supported by the following observation. The number of mutants that are not located at the most populated genotype when the latter is a local optimum is a proxy for the number of valley mutants. Its coefficient of variation increases with 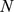 for the parameters used in fig. 5 (see fig. S5). Thus the effect of fluctuations increases with 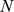 in this case, which explains why an ‘optimal’ value of 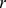 appears for 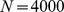 but not for 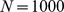.

**Figure 6:**
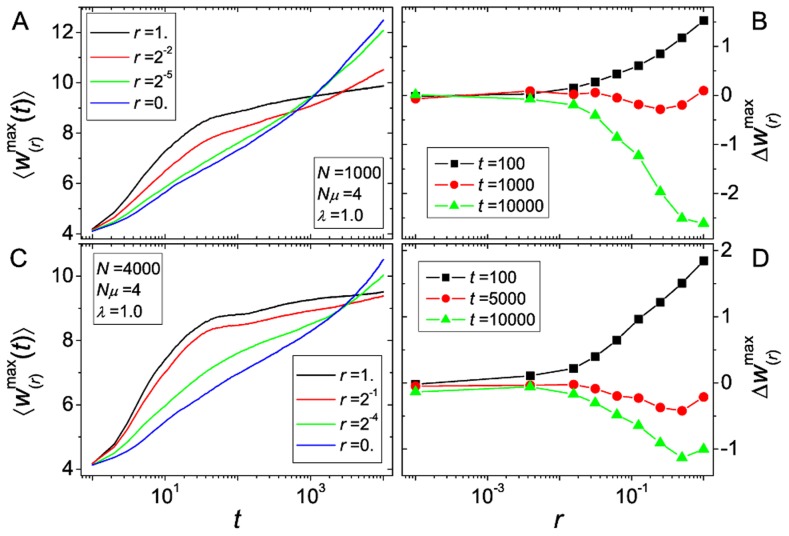
Recombinational advantage as a function of recombination rate and observation time. (A) and (C): Fitness of the fittest genotype 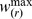 vs. time for various choices of the recombination rate 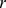. Parameters correspond to those presented in fig. 5. For short times 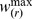 grows more rapidly for large 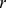 but at long times 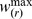 grows faster for small 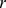. At intermediate times the acquired fitness depends non-monotonically on 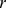. (B) and (D): 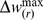 vs. 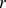 for various choices of the evaluation time 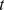. Whether high recombination rates are advantageous depends on evaluation time. At intermediate times, the dependence of recombinational advantage on 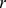 is non-monotonic. Note that for both cases shown, 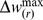 has a minimum for intermediate recombination rates at intermediate times.

It is however not clear whether the minimum in the average escape time is particularly relevant for the overall advantage of recombination. The tradeoff between long evolution times before strong trapping, as found for small 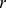, and high rates of adaptation at early times, which occur at large 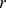, may be more relevant. A further effect that may be important at this point is a maximum of the fitness variance at intermediate 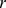 that has been found in [68] for the case of weak selection. A priori, it is difficult to predict whether this tradeoff leads to a maximal or a minimal advantage of recombination at some intermediate value of 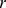. To assess these questions, we show in figs. 6A, C the temporal evolution of the mean fitness of the fittest genotype 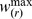 for different choices of the recombination rates, and in figs. 6B, D the value of 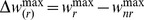 as a function of the recombination rates evaluated at a few chosen time points. For the same choices of parameters as in fig. 5, we do not observe any maximum of 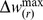 with respect to 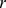 at any time. This indicates that the escape rates, which showed a maximum for intermediate 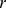, may be of minor importance, at least for the parameter values studied here.

Furthermore, fig. 6 clearly shows that whether recombination is advantageous or not depends crucially on the time at which the advantage is evaluated. For short times, at which even for high recombination rates populations did not yet get trapped, recombination is always of advantage, while for very long times high recombination rates are always disadvantageous. At intermediate times we observe a short time window for which the 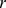-dependence of 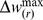 is non-monotonic. For the parameters chosen in figs. 6B, D, we observe a minimum of 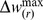 with respect to 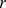, but for other parameter choices an optimal recombination rate can exist.

### Dependence on the number of loci

So far we considered landscapes with a fixed number of 16 binary loci. In this section, we are going to discuss the dependence of the dynamics on the number of loci 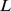. As we argued above, the existence of local maxima is crucial for the population dynamics. By increasing 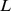, the probability of a given genotype being a local maximum, i.e., the density of local maxima, decreases. For 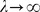 the density is known to be 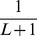 [69]. In general the density of maxima in the RMF model depends also on the distance 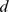 to the reference sequence, but it is always a decreasing function of 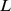 [57]. Therefore, both recombining and non-recombining populations adapt faster for larger 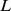 and, as we will see, the recombining ones benefit more from the thinning of local optima. However, once a recombining population is trapped, the non-recombining populations exclusively benefit from the lower trapping probability and can continue their pursuit even faster.

**Figure 7:**
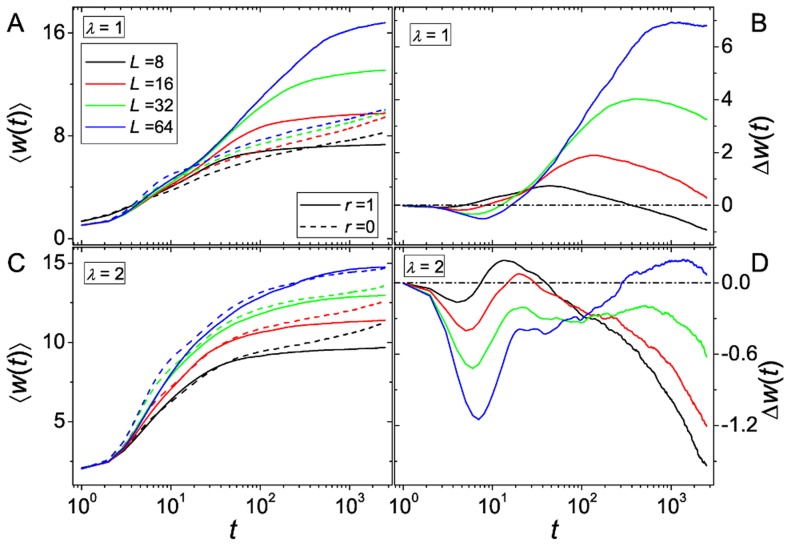
Recombinational advantage as a function of the number of loci. The figure shows time series of 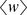 and 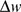, respectively, for different values of the number of loci 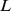. For 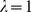 see (A) and (B), for 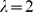 see (C) and (D). The population size is 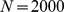 and the mutation rate is 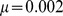.

Figure 7 shows fitness time series for different values 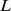. For the smaller ruggedness parameter 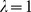, the effects that we described before are qualitatively unaltered and are just amplified due to the increase of 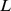. The initial decline in 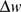 becomes more pronounced, since the ability of recombining populations to create diversity is enhanced if more neighboring genotypes are available. The intermediate time regime where recombination is advantageous is prolonged because the probability of being trapped decreases for recombining as well as non-recombining populations. The rate of fitness increase for the recombining populations increases strongly with the number of loci, in accordance with known results for additive fitness landscapes [61], [70], [71]. Hence the advantage of recombination becomes larger and lasts longer (fig. 7B).

However, when the ruggedness parameter is increased to 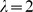, the behavior becomes more complicated. As can be seen in fig. 7C, sexual and asexual populations derive approximately the same benefit from the increased number of loci. However, the behavior of the fitness difference changes drastically. Unlike before, for larger 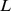 a second maximum appears in 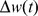, while the first maximum becomes smaller as 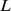 increases (cf. fig. 7D). We will discuss this phenomenon in detail in the next section. Note further that there is an advantage of recombination 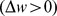 at the second maximum when 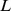 is sufficiently large. We believe that this remains true for larger ruggedness parameters, but verifying it by simulations is not feasible because it would require to increase 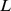 to even larger values.

At this point, it is appropriate to ask whether the phenomenology described in the previous sections will remain valid for realistically large numbers of loci, ranging in the thousands for genes or in the millions to billions for nucleotide sites. This is not immediately obvious, because the mechanism responsible for the phenomenology are the trappings at local maxima and, as we have argued before, their density decreases when increasing 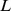. We will argue that the answer to this question depends on the ruggedness of the landscape, as quantified for the RMF model by the ratio between the ruggedness parameter 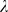 and the additive slope parameter 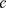 (see **Models**). It is instructive to discuss two extreme cases.

When 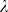 is very small compared to 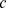 the landscape is almost additive and the population most likely reaches the global optimum. Therefore the distance a population travels before getting trapped should grow linearly with 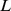. In fig. S6 we show the distance 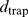 of the genotype at which the population gets trapped for the first time from 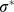, where trappings are defined as in the last section. For small 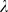 the 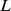-dependence of 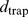 is well compatible with a linear relation (fig. S6A in the supplementary material). Therefore, in this regime, when 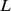 becomes large, trapping at local maxima will be very rare and should only play a minor role in the dynamics, thus invalidating the phenomenology described earlier.

At the other extreme, when 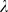 is large compared to 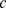, the population is likely to end up in a local optimum. Although we do not have a precise prediction for how far such a population travels before getting stuck, we can refer to the behavior in the well-studied strong selection weak mutation (SSWM) regime, which is characterized by the condition 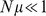 [31], [34]. In the SSWM regime the population is almost monomorphic and evolves as a single entity performing an uphill *adaptive walk* on the landscape. In the case of a completely random landscape (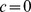), the distance a population travels before it gets stuck grows logarithmically with 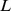 [31], [32], [34], [36], [37]. This result remains valid in the RMF model for small 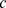 [57]. Although our dynamics takes place far from the SSWM regime, the logarithmic prediction is compatible with our numerical findings for highly rugged landscapes (fig. S6B in the supplementary material). If this relation prevails for extremely large 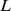, we expect the trapping at local maxima to be relevant for realistically large genomes and the mechanism leading to a disadvantage of recombination at long times to remain valid.

### Time scales and dynamic regimes

Having explained the transient nature of the advantage of recombination in qualitative terms, the next step would be to estimate the time 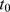 up to which recombination is, on average, of advantage, the time 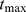 at which recombination is, again on average, of largest advantage, and the corresponding maximal advantage 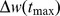. Let 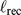 denote the mean distance a recombining population can travel before it gets stuck in a local maximum, and 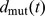 the mean distance at time 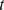 from the initial genotype 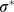 of a non-recombining population. Then 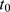 can be estimated from 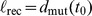. Unfortunately, we do not have analytic expressions for any of these two quantities and it seems improbable that they should be easy to obtain. The distance 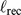 is similar to the length of adaptive walks which has been calculated for non-recombining populations in the SSWM approximation [31]–[37]. This approximation is clearly not applicable for recombining populations, as recombination requires polymorphism. Expressions for 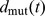 have been derived for populations evolving beyond the SSWM regime (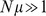), but these works are largely restricted to non-epistatic landscapes (for a review see [72]). The only exception known to us [73] employs an infinite sites limit, which eliminates the possibility of trapping at local fitness maxima.

As we argued before, the probability of trapping depends on the mean density 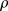 of local maxima. It therefore seems reasonable to expect that the maximal acquired recombination advantage 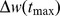 should be the larger, the smaller 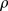. A priori, it is not clear whether this also applies to the time 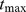 at which the maximum of 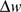 is reached, but simulations suggest that this is the case (see fig. S7).

In order to succinctly summarize the findings of the preceding sections and indicate how they can be generalized, we rephrase them in terms of the relative temporal ordering of the three different regimes of the evolutionary dynamics. These regimes are:

1. The *initial regime* where the population starts spreading from its monomorphic initial condition. Most of the fitter neighboring genotypes will be populated and this regime is marked by a large diversity.
2. The regime of *almost sequential dynamics (ASD)* where the population is quasi-monomorphic and moves by sequentially fixing at increasingly fit, mostly neighboring, genotypes. It ends when a local maximum is reached.
3. In the *final regime*, the population is trapped at local maxima most of the time. Since escape is only possible by crossing a fitness valley, adaptation becomes very slow.

**Figure 8:**
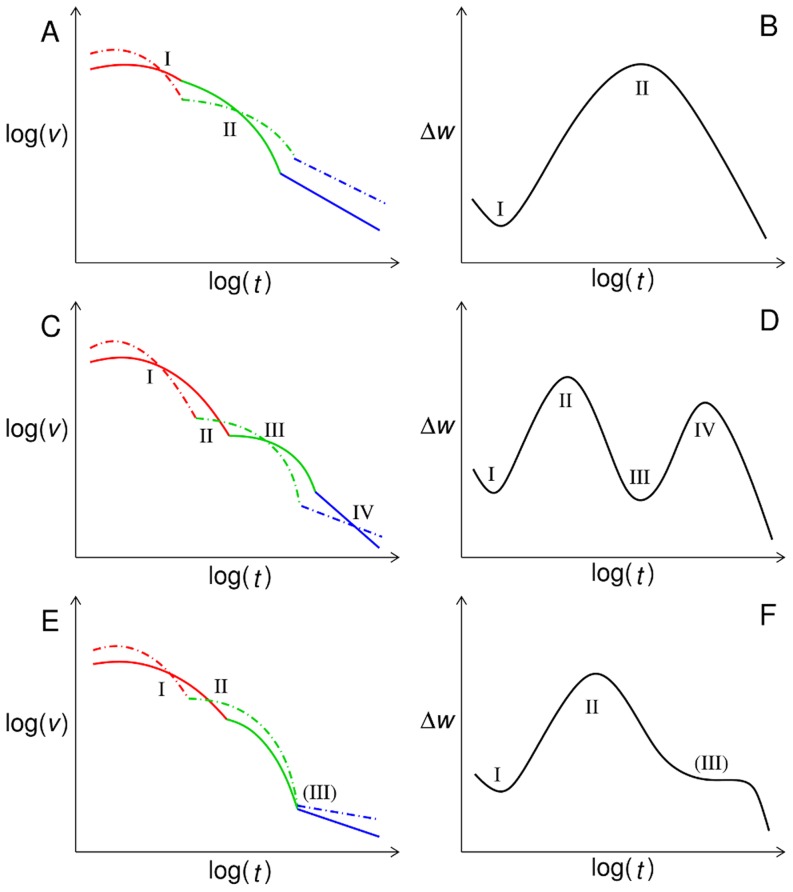
Summary of scenarios for a transient advantage of recombination. The figure shows schematic plots of fitness velocities 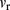 (solid lines), 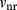 (dashed lines) (A, C, E) and the corresponding behavior of 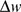 (B, D, F). Red, green and blue line segments correspond to the initial, ASD and final regime, respectively. Latin numbers indicate intersections of the velocities and the corresponding extrema in 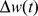. The last number in panels E and F is written in parenthesis to indicate that the two curves do not actually intersect but come very close to each other.

In practice it may be hard to clearly distinguish these regimes in an actual fitness time series. For a systematic discussion it is convenient to introduce the fitness velocities defined as 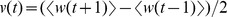, again with subscripts “r” and “nr” for recombining and non-recombining populations, respectively. The different regimes correspond to different behaviors of 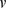. In order to explain the phenomenology we refer to a schematic picture of the velocities and the corresponding behavior of 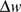 in fig. 8, for plots showing real data see fig. S8. Note that intersections of 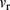 and 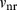 correspond to extrema in 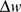. As a consequence, 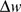 can have several maxima depending on the number of intersections. In fig. 8 we display three cases, one with a single maximum followed by a smooth decay, one case with two maxima, and finally again a case with a single maximum but with a hump in the eventual decay.

For both recombining and non-recombining populations, the velocities decay monotonically, apart from a short initial period. This mainly reflects the simple fact that, in a static fitness landscape, the availability of beneficial mutations decreases as the fitness level of the population rises [73]. This circumstance is aggravated by the structure of the RMF landscape, where after each step away from 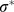 the number of uphill neighbors, which have a greater likelihood to be of higher fitness, is decreased by one [57]. In addition, as time elapses, in an increasing number of realizations the populations will end up trapped in local maxima, further decreasing the average velocity of the ensemble.

Non-recombining populations start increasing their fitness more rapidly at short times as they immediately concentrate on some particularly fit neighbors, while recombining populations keep a larger diversity that lowers their average fitness. Due to this concentration the non-recombining populations start their ASD and therefore their velocity falls off rapidly, in accordance with Fisher's fundamental theorem. Because of their higher diversity the velocity of the recombining populations falls off more slowly, allowing them to overtake the non-recombining ones at least in velocity.

When all the non-recombining populations either became trapped or entered the ASD, the decay of their average velocity slows down, while for the recombining populations the decay is still rapid. The subsequent behavior depends on whether the recombining populations on average enter their ASD regime before or after their velocities have fallen below the level of the non-recombining populations. The first case is illustrated in fig. 8A. Eventually, as all populations get trapped on local optima, the velocity of recombining populations must fall below that of non-recombining ones, giving rise to a second intersection and thus to a single maximum in 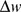 (cf. fig. 8B).

On the other hand, if recombining populations enter the ASD regime after the point where 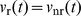, two scenarios are possible. Either, due to the deceleration when the recombining populations enter the ASD regime, a third intersection emerges which gives rise to a second maximum in 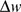 (fig. 8C, D). Or there is no third intersection, but due to the slower velocity decay, the velocities of recombining and non-recombining populations approach each other, causing a hump in the decay of 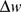 after the maximum (fig. 8E, F). Simulation data illustrating all three scenarios are displayed in fig. S8.

### Fitness seascapes

If recombination is only of transient advantage one must ask why it is nevertheless so ubiquitous. One explanation may be that recombination would preferably show up in the case of weak selection, i.e. nearly neutral fitness landscapes. In such cases, recombination can break up linkage, impeding trappings, so that the populations can be expected to behave similar to populations on smooth landscapes [53], [54].

If recombination is also ubiquitous in the presence of strong selection, there must exist mechanisms that considerably prolong the period of advantage. For example, as we have shown earlier, the larger the number of loci, the more advantageous recombination is and the longer the advantage lasts. Another mechanism that might prolong the advantageous period is disruptive selection [74], which can lead to stable coexistence of distant genotypes. This would make genetically homogeneous populations less likely and would thus reduce the probability of trappings at local optima.

Moreover, a prolonged beneficial effect of recombination can result from the genome being organized in such ways that epistasis, which may give rise to local fitness maxima, only occurs within fixed blocks of the genome which are preserved under recombination [51]. Strong trapping due to recombination can only occur at local maxima that must be escaped by combining mutations on loci located on different blocks. As in the setup proposed in [51] no such local maxima exist, the advantage of recombination should prevail forever. However, more realistically, there would be at least some epistasis also between the blocks. For large genomes the existence of local optima would then still be likely, although the times for which the population can adapt freely before getting caught in such a state may be extremely long.

Here we examine a fourth possibility to prolong the benefit of recombination. Suppose a population lives in an environment that changes rapidly in time. Then the probability for the population to survive does not depend so much on its ability to reach the fittest possible state in the long run, but rather to rapidly escape from the poorly adapted state it finds itself in after the external conditions have abruptly changed. Therefore, we expect recombination to be beneficial in the long run if the time scale at which the fitness landscape changes is shorter than the time scale at which recombination stops being beneficial on a static landscape. Such fluctuating fitness landscapes are sometimes called fitness seascapes [58]. Note that a resetting of a fitness landscape does not necessarily need to be the result of fluctuating environments, but could also be the result of a population traveling through a spatially inhomogeneous world.

In order to test our hypothesis that the beneficial effect of recombination may be sustained indefinitely in fluctuating environments we constructed seascapes as follows. At each time step of the population dynamics, the landscape is reset with probability 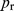, i.e., the random variables 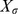 defining the random fitness components in eq. (3) are redrawn and the reference sequence 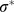 is newly chosen. The new reference sequence is either selected from one of the neighbors of the preceding one or picked completely at random. We will refer to the former selection mode as a *soft reset* and to the latter as a *hard reset*. Note that models of single-peaked fitness landscapes with a moving optimum have been considered previously [75]–[79] (see also [7], [20] and references therein).

**Figure 9:**
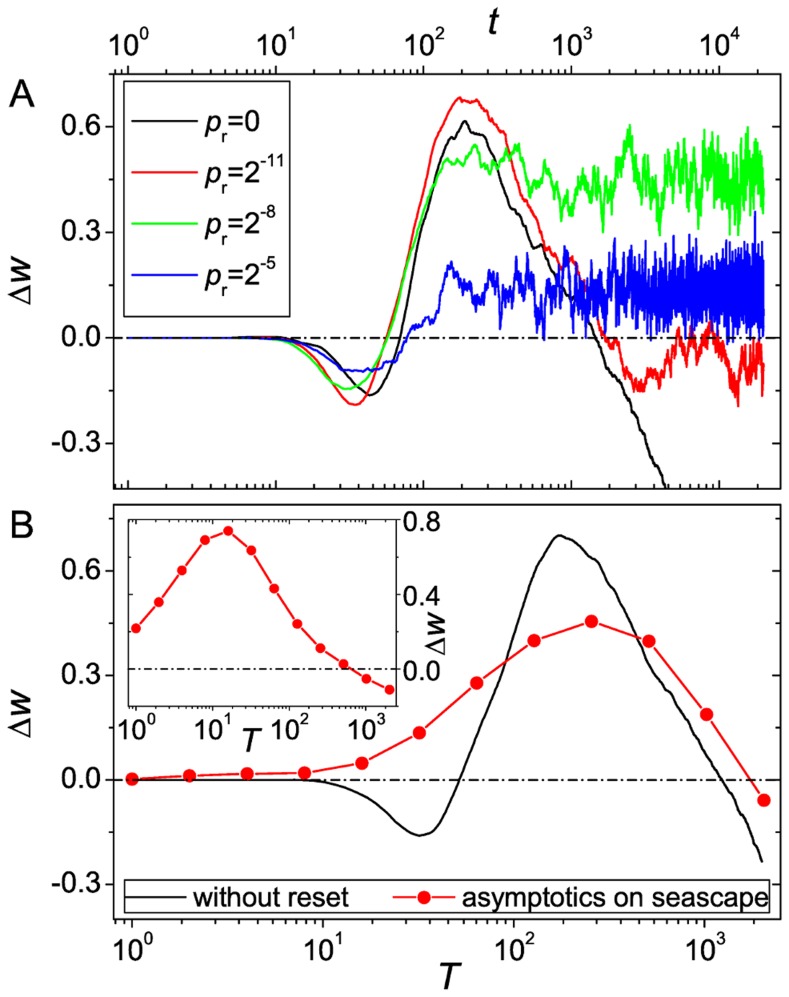
Advantage of recombination in fitness seascapes. (A) Time series of the fitness advantage 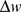 of a seascape with hard reset. (B) Asymptotic fitness advantage 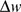 in dependence on 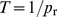 in the stationary state of the seascape with hard reset. The data are extracted from the time series and correspond to the value of 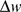 averaged over generations 5000 to 20000. For comparison we also plotted the time series for the same set of parameters on the corresponding (static) landscape and a random initial genotype. The parameters are 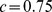, 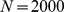 and 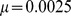. In the inset we show the stationary advantage on a seascape with soft reset.

Since local maxima only exist temporarily in this setting, the population will not be trapped forever at any specific genotype. Instead, the global maximum will be continuously pursued. In case of a soft reset, the direction of the global fitness gradient remains essentially unchanged after resetting the seascape whereas the adaptation process starts anew from a random position in case of a hard reset. In both cases the mean fitness advantage of recombination becomes stationary after some time (fig. 9A). The mean value of this stationary advantage depends on how well the population is able to follow the global optimum. Based on our results for static landscapes, it is clear that the recombining population has the largest advantage if the time 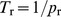 between two resets of the seascape is of the same order as the time 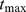 that a population on a static landscape can evolve freely before being trapped at a local maximum. Moreover, recombination becomes disadvantageous if 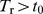, i.e., if the time between two resets is longer than the time a non-recombining population needs to overtake a recombining population.

As a consequence, the asymptotic fitness advantage 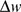 with respect to 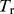 on a seascape behaves similar to the time-dependent advantage 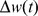 on a static landscape (fig. 9B). Using a physical analogy, we can say that the stationary advantage of recombination is maximized when the intrinsic time scale of adaptation is in resonance with respect to the environmental alteration time. The dip at short times in the time series 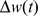 is not visible in the stationary advantage 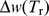. This is because, after the resetting of the landscape, the population is not monomorphic, which means that the short time regime associated with the buildup of diversity discussed previously in the context of static landscapes is absent on the seascape. Note also that the curves corresponding to the time-dependent advantage on static landscapes and the stationary advantage on the seascape coincide only if suitable initial conditions are used in the former case. For the comparison with the hard reset in fig. 9B, the initial population in the static landscape simulations was therefore placed at a randomly chosen sequence rather than at 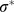.

## Discussion

In this article we have numerically examined the benefit of recombination in a class of tunably rugged fitness landscapes. A few earlier papers have been devoted to the systematic study of the effect of sign epistasis on the advantage of sex on high dimensional fitness landscapes [47]–[50], [52], but they either considered very specific landscape realizations [47]–[49] or did not take into account the temporal development of the evolutionary advantage due to recombination [50], [52]. Focusing on this temporal development, we find that the advantage of recombination is a strictly transient effect, which is particularly interesting as this observation coincides nicely with recent experiments carried out with the facultatively sexual rotifer *Brachionus calyciflorus* [80]. We note that an increased appreciation of transient, as compared to asymptotic mechanisms has also been promoted in the context of ecological theory [81].

Our analysis suggests that several of the well known effects associated with recombination play an important part in the evolutionary dynamics, but their importance differs at different timescales. At very short timescales the Weismann effect is dominant, forcing the population to distribute more broadly over the genotype space. This leads to an initial fitness disadvantage of recombination, but note that this is to some extent an artifact of the monomorphic initial condition. Such a short time disadvantage has been reported in experiments with *Saccharomyces cerevisiae* [82]. Although there the initial disadvantage of recombination was attributed to the costs of sexual reproduction which are absent in our numerical setup, it is plausible that the disadvantage is at least partly caused by the mechanism we described here.

At intermediate timescales, the Fisher-Muller effect makes use of the increased population diversity for producing distant high-fitness mutants, which leads to an advantage of recombination. This regime continues until the populations concentrate at local fitness maxima. Using known results from studies of 2-locus-models, we argue that, especially for large 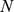, recombination prevents the escape from local optima and populations get trapped on such states for very long times. For non-recombining populations the trapping is much weaker, leading to shorter escape times. Hence they can proceed gaining fitness while recombining populations remain stuck, losing all the previously built up advantage in the long run. With an increasing number of optima, such trappings will of course occur earlier. Roughly speaking, the advantage of recombination lasts longer the less rugged the underlying landscape is.

An important question that we have discussed is how relevant such trapping events are in very large landscapes corresponding to thousands of genes or millions of nucleotides. For a state to be a local maximum, all its neighbors must be of lower fitness, which is the more improbable the larger the number of loci 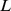. This question is not easy to address in general terms, as the answer will depend on the landscape model [24]. Referring to known results for adaptation in the SSWM regime [31], [32], [34], [36], [37], we have argued that trapping will remain relevant provided the distance travelled by an adaptive walk to a local optimum increases sufficiently slowly (logarithmically) with the number of loci, as appears to be the case for the RMF model provided the slope parameter 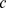 is not much larger than the ruggedness parameter 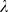 [57]. Thus the scenario proposed here remains valid for large 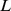 if the fitness landscape is sufficiently rugged.

The outstanding importance of the strong trapping of recombining populations has previously been noted in [52], where also the advantage of recombination on high dimensional fitness landscapes with epistasis was studied. Unlike in our study, however, the advantage of recombination was evaluated after a fixed time. The same is true for [50], where different quantities characterizing the landscape were tested with respect to their ability to predict whether recombination will be advantageous or not. Our results show that the question of whether recombination is advantageous is well defined only if the corresponding temporal scale is specified. Unless the time scale is clearly determined by the context, e.g., for a particular experimental setup or an ecological scenario, it may be more appropriate to assess the advantage of recombination through quantities like 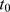, 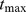 or 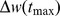 defined above.

A transitional benefit of recombination has been observed in various previous studies, both on the level of the fitness trajectories of recombining vs. non-recombining populations [61] and with regard to the temporal change of the frequency of a modifier allele governing the rate of recombination (see e.g. [4], [7]). However, these studies did not consider fitness landscapes with local maxima where populations could get trapped, and the transient nature of the recombinational advantage arose because the global maximum was found by the recombining population. This difference is important when the number of loci becomes large, because then finding the global optimum is illusive and thus irrelevant for the dynamics, while getting trapped by local maxima is still very common.

We have shown that on time dependent landscapes the advantage of recombination can subsist for indefinite times as long as the time scale on which the landscape changes is shorter than the time scale at which non-recombining populations can overtake trapped recombining ones. This finding is in concordance with van Valen's Red Queen Hypothesis [12], and it specifies the time scale of environmental change that confers the maximal long-time benefit. An indefinitely prolonged advantage of recombination on time dependent landscapes with a single moving optimum has been reported previously (see e.g. [20] and references therein). Again, our model emphasizing the importance of trapping at local optima provides a rather different perspective on this effect, because the time scale on which local optima appear and vanish can be significantly different from the dynamics of the global peak.

A number of interesting questions could not be addressed in this study. For example, this article deals only with the dynamics in limit where selection is strong compared to recombination. In [53], [54] it has been shown that in the case of weak selection, a different dynamics of recombining populations is to be expected, where recombination can break up linkage, such that trappings do not occur anymore and the populations are well described by their allele frequencies. Although one might expect that in this regime the populations behave similarly to those on smooth landscapes, this point needs to be investigated. Further, it would be important to determine in experiments which of the two regimes is the relevant one for naturally recombining populations.

Another open questions concerns the behavior of diploid populations. Considering the case of haploids, the existence of appropriate local maxima yields multiple equilibria in the 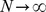 limit, where the recombining populations are strongly centered around one of the suboptimal peaks. Strictly speaking, these are not stationary states for finite 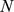, but the times that a recombining population needs to escape from such states can still be extremely long. For diploid populations in the 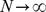 limit, on the other hand, multiple equilibria can exist even in the absence of local maxima in the genotypic fitness landscape [83]. It would therefore be interesting to investigate, whether the existence of such multiple equilibria that emerge in the absence of sign epistasis will lead to long trapping times for finite diploid populations.

Finally, it might also be important to investigate other, perhaps more realistic, recombination schemes such as single- or multi-point crossovers. Another interesting choice would be block recombination [51], which, for suitable epistatic interactions within or between blocks, has already been shown to be able to considerably prolong the time for which recombination is beneficial.

## Models

### Fitness landscape

We consider haploid populations with a binary alphabet for the representation of the genome, i.e. the genome is modeled as a binary sequence 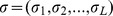 of a fixed length 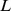. This sequence can for example be interpreted as 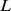 genes or nucleotides that are either identical to a wild type allele or a mutated variant. The set of genotypes is then given by the hypercube 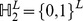. Together with the Hamming distance 
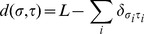
(2)between two genotypes 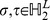 this structure becomes an 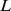-dimensional metric space, the Hamming space. In eq. (2) the Kronecker symbol equals 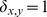 if 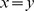 and 0 else.

To each genotype 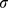 we assign a real number 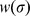 that represents the fitness associated with 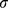. In this paper we mostly consider dynamics on landscapes created according to the Rough Mount Fuji (RMF) model [24], [55]–[57], where the fitness values are assigned as 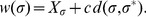
(3)


Here the 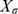 are random numbers drawn *independently* from an exponential distribution with mean 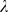, 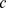 is the mean slope of the landscape, and 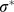 is a reference sequence. On average, the fitness values become larger with increasing distance to 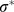. For 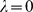 the landscape defined by (3) is purely additive, and for 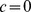 it reduces to a model where fitness values are assigned randomly and independently to genotypes [69]. Because of its similarity to a mutation-selection model introduced in [84] the latter case is often referred to as the House of Cards (HoC) model [18], [24], [39]. To test the robustness of our conclusions we present results obtained using Kauffman's NK-model [64] in fig. S3 (see [24], [25], [27] for further information about this model).

### Finite population dynamics

We use a slightly modified version of the Wright-Fisher model with constant population size. Let 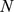 (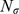) denote the number of individuals in the population (carrying genotype 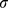) and 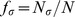 the fraction of individuals with genotype 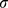. A single time step of the population dynamics consists of the following substeps:

**Mutation.** On each genotype 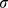, a fraction 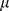 of individuals mutates to the neighboring genotypes: 
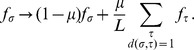



**Selection.** The fraction of individuals on each genotype is decreased or increased, depending on the fitness value compared to the average fitness: 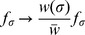
where 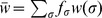.

**Random sampling.** On each genotype 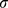, the number of individuals is replaced by a random variable drawn from a Poisson distribution with mean value 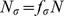. After this process, the population size is actually 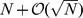, such that we have to normalize the population in order to keep it constant. Note that usually a multinomial distribution on all genotypes at once is used instead of independent Poisson distributions. However, we chose this method because it makes the simulation much faster [85].

**Recombination.** A fraction 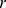 of individuals is replaced by other individuals whose genotypes are recombinants of two randomly chosen parents. Recombination is performed with a uniform crossover scheme, i.e., each locus is either taken from the first or the second parent with equal probability. If 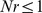, the number of individuals which is replaced is drawn from a Poisson distribution with mean value 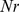.

### Infinite population dynamics

The infinite population size limit results in a deterministic dynamics on the set of genotype frequencies 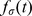 [20]. For convenience we employ here an algebraic formulation, which is explained in the following.

A single mutation can only transfer a sequence into one of its neighbors. The *adjacency matrix* encodes the information about the neighborhood structure of the hypercube, and is defined by 
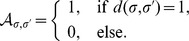



Considering an evolutionary dynamics with mutation probability 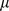, at every time step a fraction 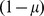 of the population with a given genotype does not mutate, while a fraction 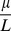 mutates to each of its 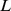 neighbors. Thus, the single step mutation matrix is defined by 
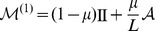
where 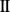 denotes the identity operator. As 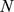 gets larger, double mutations that appear with a rate of order 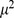 become more frequent and must be taken into account. The mutation matrix including two step mutation events is 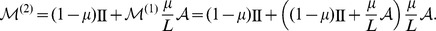



In the same manner, this can be generalized to include mutations up to 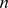
–th order: 
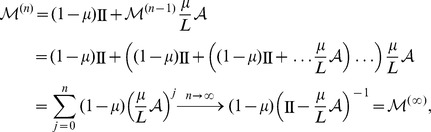
where the limit is performed using the geometric series of matrices, which exists because 
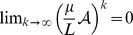
. Note that for infinite populations mutations of no order can be neglected and, thus, 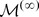 is the appropriate mutation matrix in this limit.

Recombination still only happens once per generation, thus it is realized by the corresponding transition matrix 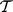 defined in [86]. The selection matrix is 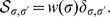



Then the genotype frequencies 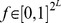 evolve in time according to 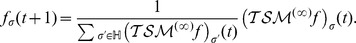



This means in particular, that already after the first time step every genotype is created by mutation. Starting with a monomorphic population, 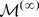 leads to a distribution of the population that decays exponentially with the Hamming distance to the initially populated state. This can cause numerical problems if the numbers representing the population become too small. In order to be sure that the population is not neglected on any state of the landscape at any time due to limits of computational precision, the simulations were performed with a precision of more than 30 significant digits and we restrict ourselves to small numbers of loci.

## Supporting Information

Figure S1Comparison of 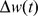, 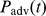 and the frequency of recombining individuals for the case with a modifier allele 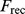 as quantifiers for the (dis-)advantage of recombination. Parameters are the same as in fig. 2. All quantities are correlated and show qualitatively the same behavior. Nevertheless, there are regions on the time axis where 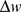 shows a recombination advantage while 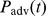 indicates a disadvantage and vice versa. This implies that the distribution of 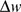 is not centered around the mean value, or more precisely, the mean value is not equal to the median.(PDF)

Figure S2Comparison of the entropy 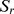 and the additive genetic variance 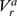 (A) as well as 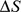 and 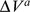 (B). Parameters are the same as in fig. 2. All quantities are correlated and show qualitatively the same behavior.(PDF)

Figure S3Transitory advantage of recombination on Kauffman's NK-landscape with 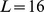 binary loci. Each locus interacts with 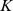 adjacent neighbors, and fitness values are drawn from a lognormal distribution. Population parameters are 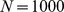 and 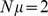. For increasing ruggedness, i.e., for increasing values of 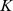, the advantage becomes less pronounced and vanishes. Data marked ‘HoC’ correspond to the maximally rugged case 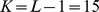, where the NK-model reduces to the House of Cards model with uncorrelated random fitness values.(PDF)

Figure S4Dependence of the recombinational advantage on population size. Same as fig. 3. but with constant 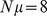 and varying population size 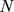.(PDF)

Figure S5The coefficient of variation 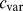 of the number of mutants that are not located at the most populated genotype when the latter is a local optimum vs. 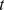 for systems with 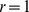, 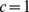, and 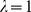. Note that, for constant 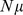, 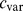 increases with 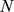.(PDF)

Figure S6Trapping distance increases with landscape dimensionality. The figure shows the distance 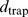 at which populations are trapped for the first time vs. number of loci 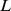 for 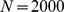, 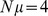, 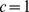, and (A) 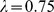, (B) 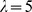. Straight lines are guides to the eye and show that the 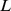-dependencies are well compatible with a (A) linear and (B) logarithmic relation, respectively.(PDF)

Figure S7Recombinational advantage declines with the density of local maxima. To clarify whether the time of maximal recombination advantage, 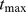, increases with decreasing density of local maxima, 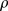, we measured 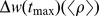 and 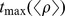 in simulations, where 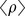 is the mean density of maxima averaged over realizations as well as over genotype space (recall that the density of maxima is inhomogeneous in the RMF model). Panel (A) shows 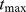 and panel (B) the corresponding maximal advantage 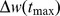 vs. 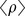. Both quantities decline monotonically with increasing 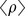. The density was controlled by changing the slope 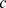 for fixed 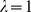 (see inset of panel (A)). Data correspond to 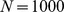, 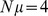 and 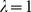.(PDF)

Figure S8(A), (C), and (E): Fitness velocities vs. 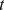 for systems with parameters 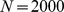, 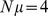, and 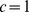. Intersections are marked by dashed lines. (B), (D), and (F): Corresponding curve for 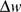 vs. 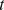. Compare with curves in the schematic pictures in fig. 8.(PDF)

## References

[1] Butterfield NJ (2000) *Bangiomorpha pubescens* n. gen., n. sp.: implications for the evolution of sex, multicellularity, and the Mesoproterozoic/Neoproterozoic radiation of eukaryotes. Paleobiology 26: 386–404.

[2] Maynard Smith J (1978) The Evolution of Sex. Cambridge, UK: Cambridge University Press.

[3] Michod RE, Levin BR (1987) The Evolution of Sex: An Examination of Current Ideas. Michigan: Sinauer Associates. 352 p.

[4] Feldman MW, Otto SP, Christiansen FB (1997) Population genetic perspectives on the evolution of recombination. Annu Rev Genet 30: 261–295. 8982456 10.1146/annurev.genet.30.1.261

[5] Otto SP, Lenormand T (2002) Resolving the paradox of sex and recombination. Nat Rev Gen 3: 252–261. 10.1038/nrg761 11967550

[6] de Visser JAGM, Elena SF (2007) The evolution of sex: empirical insights into the roles of epistasis and drift. Nat Rev Gen 8: 139–149. 10.1038/nrg1985 17230200

[7] Otto SP (2009) The evolutionary enigma of sex. Amer Nat 174: S1–S14. 19441962 10.1086/599084

[8] Charlesworth B, Charlesworth D (1975) An experiment on recombination load in Drosophila melanogaster. Genet Res 25: 267–274. 810390 10.1017/s001667230001569x

[9] Muller HJ (1964) The relation of recombination to mutational advance. Mutat Res 106: 2–9. 14195748 10.1016/0027-5107(64)90047-8

[10] Felsenstein J (1974) The Evolutionary Advantage of Recombination. Genetics 78: 737–756. 4448362 10.1093/genetics/78.2.737 PMC1213231

[11] Kondrashov AS (1988) Deleterious mutations and the evolution of sexual reproduction. Nature 336: 435–440. 3057385 10.1038/336435a0

[12] Van Valen L (1973) A new evolutionary law. Evol Theor 1: 1–30.

[13] Fisher RA (1930) The genetical theory of natural selection. Oxford: Oxford Claredon Press.

[14] Muller HJ (1932) Some genetic aspects of sex. Amer Nat 66: 118–138.

[15] Hill WG, Robertson A (1966) The effect of linkage on limits to artificial selection. Genet Res 8: 269–294. 5980116

[16] Weismann A (1889) Essays on heredity and kindred biological subjects. Oxford Univ. Press, Oxford, UK.

[17] Wright S (1932) The roles of mutation, inbreeding, crossbreeding and selection in evolution. Proc of the 6th Int Cong of Genetics 1: 356–366.

[18] de Visser JAGM, Krug J (2014) Empirical fitness landscapes and the predictability of evolution. Nat Rev Gen 15: 480–490. 10.1038/nrg3744 24913663

[19] Provine WB (1986) Sewall Wright and Evolutionary Biology. Chicago: University of Chicago Press.

[20] Bürger R (2000) The Mathematical Theory of Selection, Recombination, and Mutation. New York: JohnWiley& Sons, Ltd.

[21] de Visser JAGM, Cooper TF, Elena SF (2011) The causes of epistasis. Proc R Soc London B 278: 3617–3624. 10.1098/rspb.2011.1537 PMC3203509 21976687

[22] Weinreich DM, Watson RA, Chao L (2005) Perspective: Sign epistasis and genetic constraint on evolutionary trajectories. Evolution 59: 1165–1174. 16050094

[23] Poelwijk FJ, Kiviet DJ, Weinreich DM, Tans SJ (2007) Empirical fitness landscapes reveal accessible evolutionary paths. Nature 445: 383–386. 17251971 10.1038/nature05451

[24] Franke J, Klözer A, de Visser JAGM, Krug J (2011) Evolutionary accessibility of mutational pathways. PLoS Comput Biol 7: e1002134. 21876664 10.1371/journal.pcbi.1002134 PMC3158036

[25] Franke J, Krug J (2012) Evolutionary accessibility in tunably rugged fitness landscapes. J Stat Phys 148: 705–722.

[26] Nowak S, Krug J (2013) Accessibility percolation on n-trees. EPL 101: 66004.

[27] Schmiegelt B, Krug J (2014) Evolutionary accessibility of modular fitness landscapes. J Stat Phys 154: 334–355.

[28] Whitlock MC, Phillips PC, Moore FB-G, Tonsor SJ (1995) Multiple fitness peaks and epistasis. Annu Rev Ecol Syst 26: 601–629.

[29] Poelwijk FJ, Tănase-Nicola S, Kiviet DJ, Tans SJ (2010) Reciprocal sign epistasis is a necessary condition for multi-peaked fitness landscapes. J Theor Biol 272: 141–144. 21167837 10.1016/j.jtbi.2010.12.015

[30] Crona K, Greene D, Barlow M (2013) The peaks and geometry of fitness landscapes. J Theor Biol 317: 1–10. 23036916 10.1016/j.jtbi.2012.09.028 PMC3529755

[31] Gillespie JH (1983) A simple stochastic gene substitution model. Theor Popul Biol 23: 202–215. 6612632 10.1016/0040-5809(83)90014-x

[32] Flyvbjerg H, Lautrup B (1992) Evolution in a rugged fitness landscape. Phys Rev A 46: 6714–6723. 9907980 10.1103/physreva.46.6714

[33] Orr HA (2002) The population genetics of adaptation: the adaptation of DNA sequences. Evolution 56: 1317–1330. 12206234 10.1111/j.0014-3820.2002.tb01446.x

[34] Orr HA (2003) A Minimum on the Mean Number of Steps Taken in Adaptive Walks. J Theor Biol 220: 241–247. 12468295 10.1006/jtbi.2003.3161

[35] Joyce P, Rokyta DR, Beisel CJ, Orr HA (2008) A general extreme value theory model for the adaptation of DNA sequences under strong selection and weak mutation. Genetics 180: 1627–1643. 18791255 10.1534/genetics.108.088716 PMC2581963

[36] Neidhart J, Krug J (2011) Adaptive walks and extreme value theory. Phys Rev Lett 107: 178102. 22107587 10.1103/PhysRevLett.107.178102

[37] Jain K (2011) Number of adaptive steps to a local fitness peak. EPL 96: 58006.

[38] Weinreich DM, Delaney NF, DePristo MA, Hartl DL (2006) Darwinian evolution can follow only very few mutational paths to fitter proteins. Science 312: 111–114. 16601193 10.1126/science.1123539

[39] Szendro IG, Schenk MF, Franke J, Krug J, de Visser JAGM (2013) Quantitative analyses of empirical fitness landscapes. J Stat Mech Theor Exp 2013: P01005.

[40] Neidhart J, Szendro IG, Krug J (2013) Exact Results for Amplitude Spectra of Fitness Landscapes. J Theor Biol 332: 218–227. 23685065 10.1016/j.jtbi.2013.05.002

[41] Weinreich DM, Lan Y, Wily CS, Heckendorn RB (2013) Should evolutionary geneticists worry about higher-order epistasis? Curr Opin Gen Dev 23: 700–707. 10.1016/j.gde.2013.10.007 PMC4313208 24290990

[42] Weinreich DM, Chao L (2005) Rapid evolutionary escape by large populations from local fitness peaks is likely in nature. Evolution 59: 1175–1182. 16050095

[43] Weissman DB, Feldman MW, Fisher DS (2010) The rate of fitness-valley crossing in sexual populations. Genetics 186: 1389–1410. 20923976 10.1534/genetics.110.123240 PMC2998319

[44] Altland A, Fischer A, Krug J, Szendro IG (2011) Rare events in population genetics: stochastic tunneling in a two-locus model with recombination. Phys Rev Lett 106: 088101. 21405603 10.1103/PhysRevLett.106.088101

[45] Jain K (2010) Time to fixation in the presence of recombination. Theor Popul Biol 77: 23–31. 19843476 10.1016/j.tpb.2009.10.005

[46] Park S-C, Krug J (2011) Bistability in two-locus models with selection, mutation, and recombination. J Math Biol 62: 763–788. 20617437 10.1007/s00285-010-0352-x

[47] Kondrashov FA, Kondrashov AS (2001) Multidimensional epistasis and the disadvantage of sex. Proc Natl Acad Sci USA 98: 12089–12092. 11593020 10.1073/pnas.211214298 PMC59772

[48] Watson RA, Wakeley J (2005) Multidimensional epistasis and the advantage of sex. The 2005 IEEE Congress on Evolutionary Computation 3: 2792–2799.

[49] de Visser JAGM, Park S-C, Krug J (2009) Exploring the Effect of Sex on Empirical Fitness Landscapes. Amer Nat 174: S15–S30. 19456267 10.1086/599081

[50] Misevic D, Kouyos RD, Bonhoeffer S (2009) Predicting the Evolution of Sex on Complex Fitness Landscapes. PLoS Comput Biol 5(9): e1000510. 19763171 10.1371/journal.pcbi.1000510 PMC2734178

[51] Watson RA, Weinreich DM, Wakeley J (2011) Genome Structure and the Benefit of Sex. Evolution 65: 523–536. 21029076 10.1111/j.1558-5646.2010.01144.x

[52] Moradigaravand D, Engelstädter J (2012) The Effect of Bacterial Recombination on Adaptation on Fitness Landscapes with Limited Peak Accessibility. PLoS Comput Biol 8(10): e1002735. 23133344 10.1371/journal.pcbi.1002735 PMC3487459

[53] Neher RA, Shraiman BI (2009) Competition between recombination and epistasis can cause a transition from allele to genotype selection. Proc Natl Acad Sci USA 106: 6866–6871. 19366665 10.1073/pnas.0812560106 PMC2672512

[54] Neher RA, Vucelja M, Mezard M, Shraiman BI (2013) Emergence of clones in sexual populations. J Stat Mech Theor Exp: P01008

[55] Aita T, Uchiyama H, Inaoka T, Nakajima M, Kokubo T, Husimi Y (2000) Analysis of a local fitness landscape with a model of the rough Mt. Fuji-type landscape: application to prolyl endopeptidase and thermolysin. Biopolymers 54: 64–79. 10799982 10.1002/(SICI)1097-0282(200007)54:1<64::AID-BIP70>3.0.CO;2-R

[56] Aita T, Husimi Y (2000) Adaptive walks by the fittest among finite random mutants on a Mt. Fuji-type fitness landscape II. Effect of small non-additivity. J Math Biol 41: 207–231. 11072756 10.1007/s002850000046

[57] Neidhart J, Szendro IG, Krug J (2014) Adaptation in tunably rugged fitness landscapes: The rough Mount Fuji model. Genetics. E-pub ahead of print. Available: http://arxiv.org/abs/1402.3065. Accessed 25 August 2014. 10.1534/genetics.114.167668 PMC4196622 25123507

[58] Mustonen V, Lässig M (2009) From fitness landscapes to seascapes: non-equilibrium dynamics of selection and adaptation. Trends Genet 25: 111–119. 19232770 10.1016/j.tig.2009.01.002

[59] Nei M (1967) Modification of linkage intensity by natural selection Genetics. 57: 625–641. 10.1093/genetics/57.3.625 PMC1211753 5583732

[60] Nagylaki T (1993) The Evolution of Multilocus Systems Under Weak Selection. Genetics 134: 627–647. 8325492 10.1093/genetics/134.2.627 PMC1205503

[61] Kim Y, Orr HA (2005) Adaptation in Sexuals vs. Asexuals: Clonal Interference and the Fisher-Muller Model. Genetics 171(3): 1377–1386. 16020775 10.1534/genetics.105.045252 PMC1456838

[62] Feldman MW, Liberman U (1986) An evolutionary reduction principle for genetic modifiers. Proc Matl Acad Sci 83: 4824–4827. 10.1073/pnas.83.13.4824 PMC323834 3460074

[63] Barton NH (2010) Genetic linkage and natural selection. Phil Trans R Soc B 365: 2559–2569. 20643746 10.1098/rstb.2010.0106 PMC2935107

[64] Kauffman S, Weinberger E (1989) The NK Model of rugged fitness landscapes and its application to the maturation of the immune response. J Theor Biol 141: 211–245. 2632988 10.1016/s0022-5193(89)80019-0

[65] Weissman DB, Desai MM, Fisher DS, Feldman MW (2009) The rate at which asexual populations cross fitness valleys. Theor Popul Biol 75: 286–300. 19285994 10.1016/j.tpb.2009.02.006 PMC2992471

[66] Feldman MW (1971) Equilibrium studies of two locus haploid populations with recombination. Theor Pop Biol 2: 299–318. 5170718 10.1016/0040-5809(71)90022-0

[67] Rutschman DA (1994) Dynamics of the two-locus haploid model. Theor Pop Biol 45: 167–176.

[68] Turelli M, Barton NH (1990) Dynamics of Polygenic Characters under Selection. Theor Popul Biol 38: 1–57.

[69] Kauffman S, Levin S (1987) Towards a general theory of adaptive walks on rugged landscapes. J Theor Biol 128: 11–45. 3431131 10.1016/s0022-5193(87)80029-2

[70] Maynard Smith J (1971) What use is sex? J Theor Biol 30: 319–335. 5548029 10.1016/0022-5193(71)90058-0

[71] Park S-C, Krug J (2013) Rate of adaptation in sexuals and asexuals: A solvable model for the Fisher-Muller effect. Genetics 195: 941–955. 23979572 10.1534/genetics.113.155135 PMC3813875

[72] Park S-C, Simon D, Krug J (2010) The speed of evolution in large asexual populations. J Stat Phys 138: 381–410.

[73] Park S-C, Krug J (2008) Evolution in random fitness landscapes: the infinite sites model. J Stat Mech Exp Theo: P04014.

[74] Rueffler C, Van Dooren TJM, Leimar O, Abrams PA (2006) Disruptive selection and then what? Trends Ecol Evol 21: 238–245. 16697909 10.1016/j.tree.2006.03.003

[75] Maynard Smith J (1988) Selection for recombination in a polygenic model - the mechanism. Genet Res 51: 59–63. 3366381 10.1017/s0016672300023958

[76] Charlesworth B (1993) Directional selection and the evolution of sex and recombination. Genet Res 61: 205–224. 8365658 10.1017/s0016672300031372

[77] Kondrashov AS, Yampolsky LY (1996) High genetic variability under the balance between symmetric mutation and fluctuating stabilizing selection. Genet Res 68: 157–164.

[78] Nilsson M, Snoad N (2002) Optimal mutation rates in dynamic environments. Bull Math Biol 64: 1033–1043. 12508529 10.1006/bulm.2002.0314

[79] Wilke CO, Forster R, Novella IS (2006) Quasispecies in Time-Dependent Environments. Curr Top Microbiol Immun 299: 33–50. 10.1007/3-540-26397-7_2 16568895

[80] Becks L, Agrawal AF (2012) The Evolution of Sex Is Favoured During Adaptation to New Environments. PLoS Biol 10(5): e1001317. 22563299 10.1371/journal.pbio.1001317 PMC3341334

[81] Hastings A (2004) Transients: the key to long-term ecological understanding? Trends Ecol Evol 19: 39–45. 16701224 10.1016/j.tree.2003.09.007

[82] Greig D, Borts RH, Louis EJ (1998) The effect of sex on adaptation to high temperature in heterozygous and homozygous yeast. Proc R Soc Lond B 265: 1017–1023. 10.1098/rspb.1998.0393 PMC1689156 9675910

[83] Bürger R (1989) Linkage and the Maintenance of Heritable Variation by Mutation-Selection Balance. Genetics 121: 175–184. 2917713 10.1093/genetics/121.1.175 PMC1203600

[84] Kingman FC (1978) A simple model for the balance between selection and mutation. J Appl Prob 15: 1–12.

[85] Zanini F, Neher RA (2012) FFPopSim: an efficient forward simulation package for the evolution of large populations. Bioinformatics 28: 3332–3333. 23097421 10.1093/bioinformatics/bts633 PMC3519462

[86] Stadler PF, Wagner GP (1998) Algebraic Theory of Recombination Spaces. Evol Comput 5(3): 241–75. 10021760 10.1162/evco.1997.5.3.241

