# Supplementary material for: Multidimensional Epistasis and the Transitory Advantage of Sex
Source: PLoS Comput Biol. 2014 Sep 18;10(9):e1003836. doi: 10.1371/journal.pcbi.1003836 (PMC4168978; doi:10.1371/journal.pcbi.1003836)
Supplement: Figure S1 — Comparison of , and the frequency of recombining individuals for the case with a modifier allele as quantifiers for the (dis-)advantage of recombination. Parameters are the same as in fig. 2. All quantities are correlated and show qualitatively the same behavior. Nevertheless, there are regions on the time axis where shows a recombination advantage while indicates a disadvantage and vice versa. This implies that the distribution of is not centered around the mean value, or more precisely, the mean value is not equal to the median. (PDF) [file pcbi.1003836.s001.pdf]

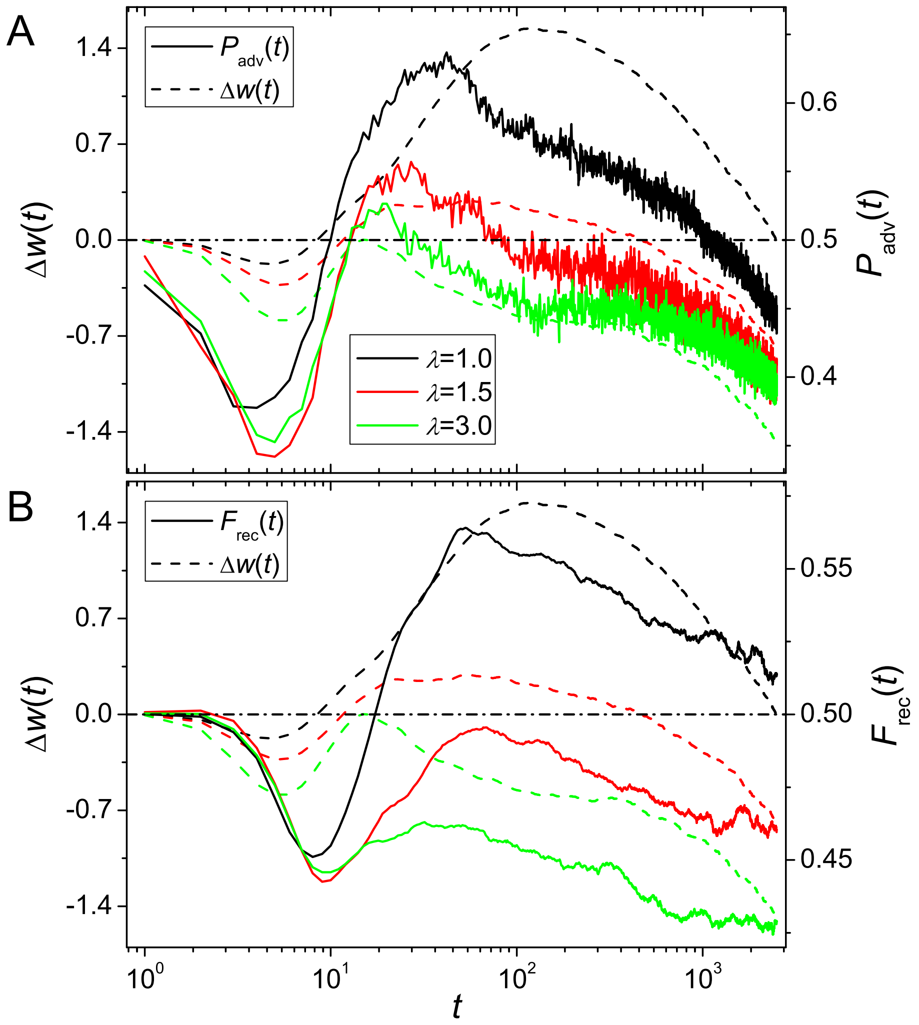


**Figure S1.** Comparison of $\Delta w(t)$, $P_{\rm adv}(t)$ and the frequency of recombining individuals for the case with a modifier allele $F_{\rm rec}$ as quantifiers for the (dis-)advantage of recombination. Parameters are the same as in fig. 2. All quantities are correlated and show qualitatively the same behavior. Nevertheless, there are regions on the time axis where $\Delta w$ shows a recombination advantage while $P_{\rm adv}(t)$ indicates a disadvantage and vice versa. This implies that the distribution of $\Delta w$ is not centered around the mean value, or more precisely, the mean value is not equal to the median.
