# Supplementary material for: Multidimensional Epistasis and the Transitory Advantage of Sex
Source: PLoS Comput Biol. 2014 Sep 18;10(9):e1003836. doi: 10.1371/journal.pcbi.1003836 (PMC4168978; doi:10.1371/journal.pcbi.1003836)
Supplement: Figure S2 — Comparison of the entropy and the additive genetic variance (A) as well as and (B). Parameters are the same as in fig. 2. All quantities are correlated and show qualitatively the same behavior. (PDF) [file pcbi.1003836.s002.pdf]

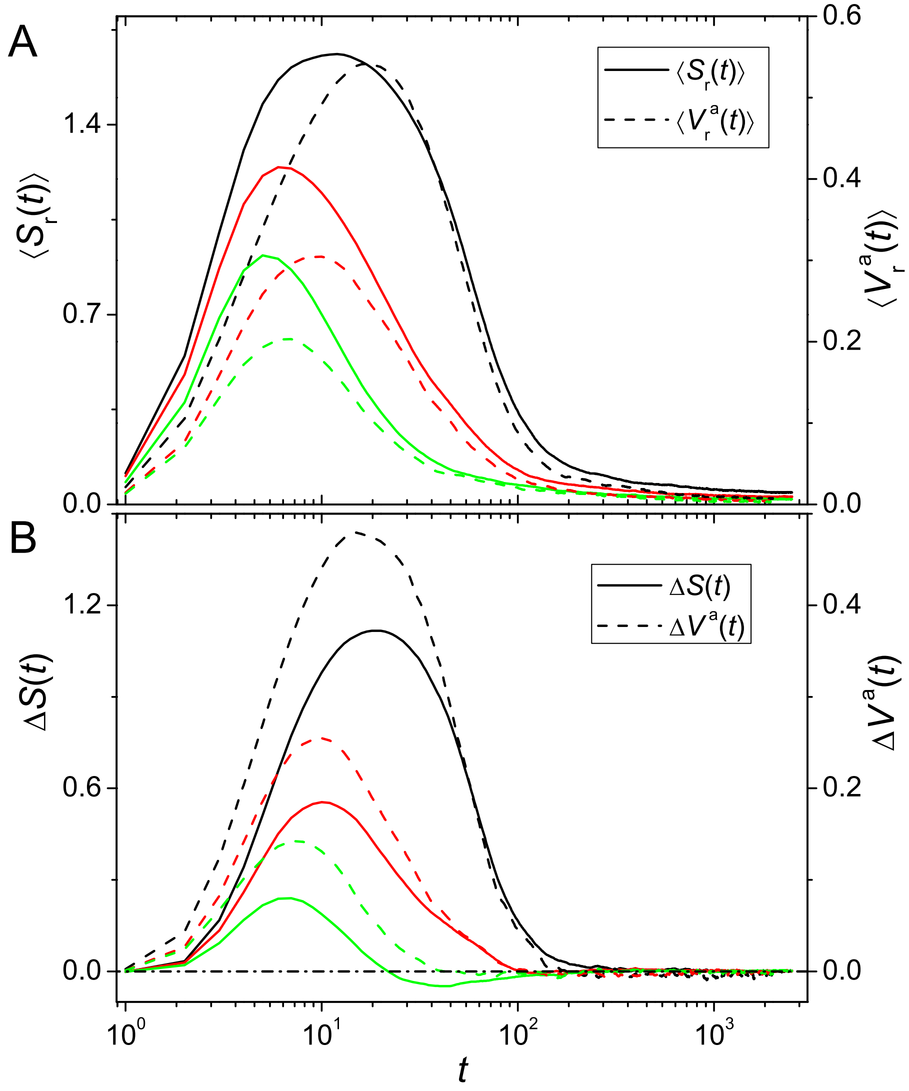

Figure S2. Comparison of the entropy S_r and the additive genetic variance V_r^a (A) as well as ΔS and ΔV^a (B). Parameters are the same as in fig. 2. All quantities are correlated and show qualitatively the same behavior.
