# Supplementary material for: Multidimensional Epistasis and the Transitory Advantage of Sex
Source: PLoS Comput Biol. 2014 Sep 18;10(9):e1003836. doi: 10.1371/journal.pcbi.1003836 (PMC4168978; doi:10.1371/journal.pcbi.1003836)
Supplement: Figure S3 — Transitory advantage of recombination on Kauffman's NK-landscape with binary loci. Each locus interacts with adjacent neighbors, and fitness values are drawn from a lognormal distribution. Population parameters are and . For increasing ruggedness, i.e., for increasing values of , the advantage becomes less pronounced and vanishes. Data marked ‘HoC’ correspond to the maximally rugged case , where the NK-model reduces to the House of Cards model with uncorrelated random fitness values. (PDF) [file pcbi.1003836.s003.pdf]

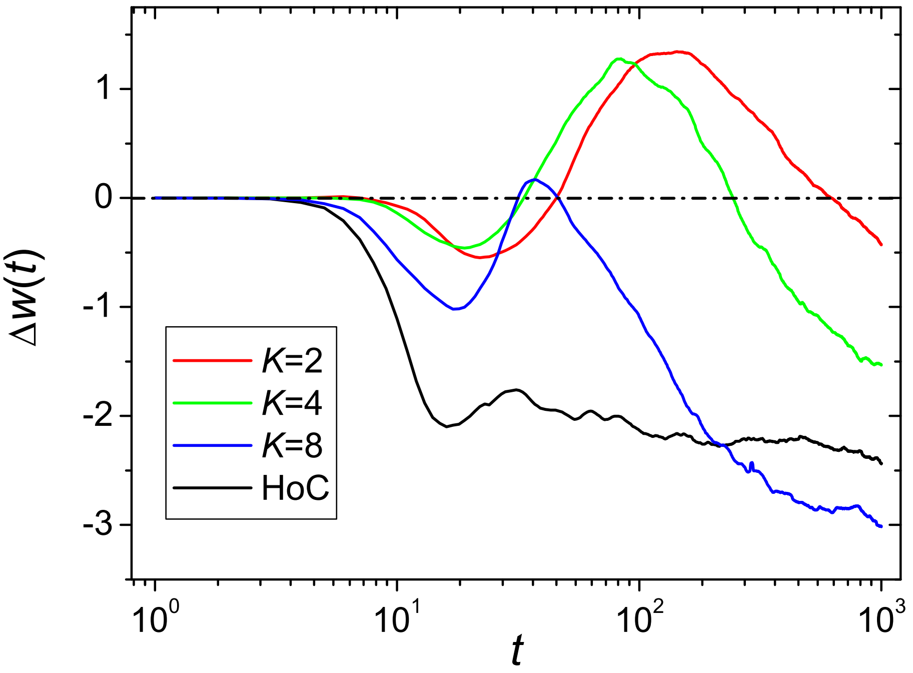

Figure S3. Transitory advantage of recombination on Kauffman's NK-landscape with $L = 16$ binary loci. Each locus interacts with K adjacent neighbors, and fitness values are drawn from a lognormal distribution. Population parameters are $N = 1000$ and $N\mu = 2$. For increasing ruggedness, i.e., for increasing values of K, the advantage becomes less pronounced and vanishes. Data marked 'HoC' correspond to the maximally rugged case $K = L - 1 = 15$, where the NK-model reduces to the House of Cards model with uncorrelated random fitness values.
