# Supplementary material for: Multidimensional Epistasis and the Transitory Advantage of Sex
Source: PLoS Comput Biol. 2014 Sep 18;10(9):e1003836. doi: 10.1371/journal.pcbi.1003836 (PMC4168978; doi:10.1371/journal.pcbi.1003836)
Supplement: Figure S4 — Dependence of the recombinational advantage on population size. Same as fig. 3. but with constant and varying population size . (PDF) [file pcbi.1003836.s004.pdf]

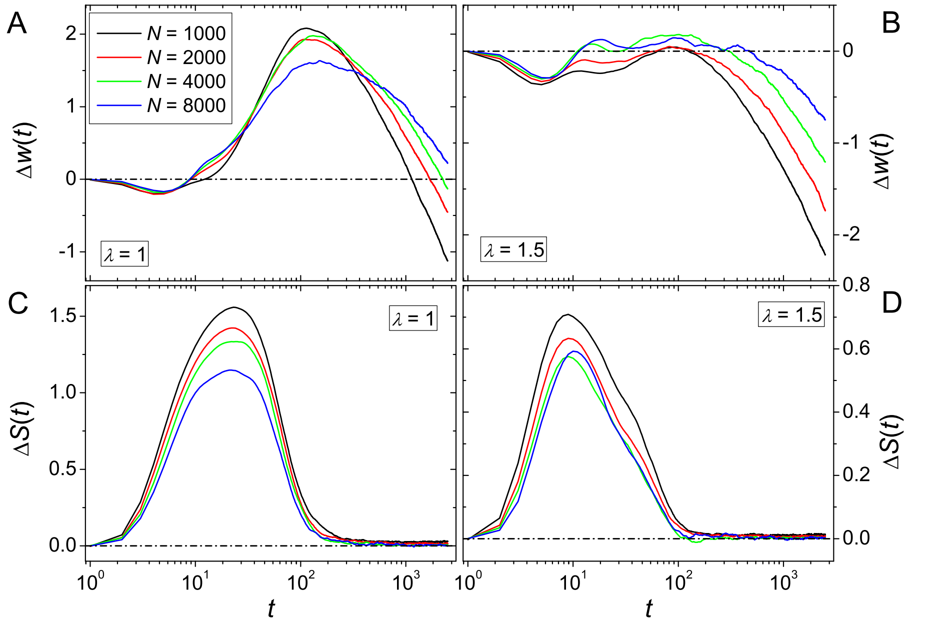

Figure S4. Dependence of the recombinational advantage on population size. Same as figure 3 but with constant $N\mu = 8$ and varying population size N.
