# Supplementary material for: Multidimensional Epistasis and the Transitory Advantage of Sex
Source: PLoS Comput Biol. 2014 Sep 18;10(9):e1003836. doi: 10.1371/journal.pcbi.1003836 (PMC4168978; doi:10.1371/journal.pcbi.1003836)
Supplement: Figure S5 — The coefficient of variation of the number of mutants that are not located at the most populated genotype when the latter is a local optimum vs. for systems with , , and . Note that, for constant , increases with . (PDF) [file pcbi.1003836.s005.pdf]

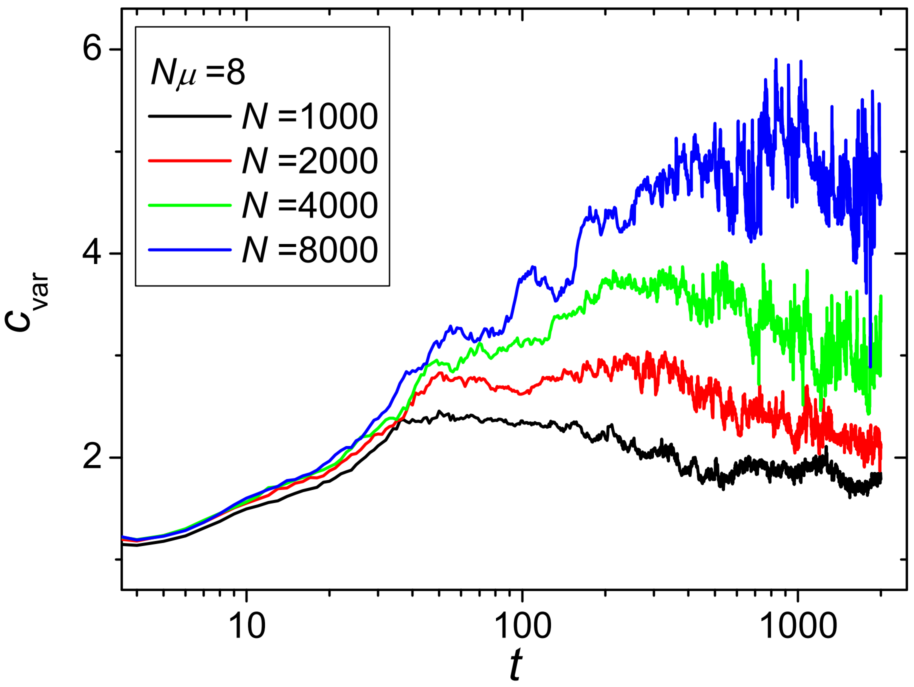

Figure S5. The coefficient of variation $c_{\rm var}$ of the number of mutants that are not located at the most populated genotype when the latter is a local optimum vs. t for systems with $r = 1$, $c = 1$, and $\lambda = 1$. Note that, for constant $N\mu$, $c_{\rm var}$ increases with N.
