# Supplementary material for: Multidimensional Epistasis and the Transitory Advantage of Sex
Source: PLoS Comput Biol. 2014 Sep 18;10(9):e1003836. doi: 10.1371/journal.pcbi.1003836 (PMC4168978; doi:10.1371/journal.pcbi.1003836)
Supplement: Figure S6 — Trapping distance increases with landscape dimensionality. The figure shows the distance at which populations are trapped for the first time vs. number of loci for , , , and (A) , (B) . Straight lines are guides to the eye and show that the -dependencies are well compatible with a (A) linear and (B) logarithmic relation, respectively. (PDF) [file pcbi.1003836.s006.pdf]

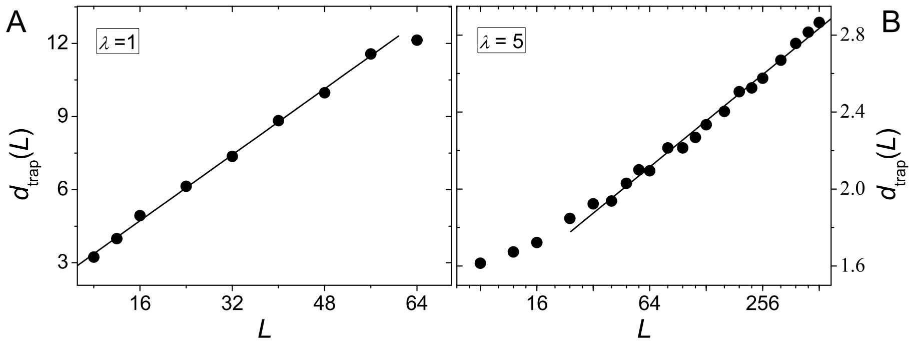

Figure S6. Trapping distance increases with landscape dimensionality. The figure shows the distance d_{trap} at which populations are trapped for the first time vs. number of loci L for $N = 2000$, $N\mu = 4$, $c = 1$, and (A) $\lambda = 0.75$, (B) $\lambda = 5$. Straight lines are guides to the eye and show that the L-dependencies are well compatible with a (A) linear and (B) logarithmic relation, respectively.
