# Supplementary material for: Multidimensional Epistasis and the Transitory Advantage of Sex
Source: PLoS Comput Biol. 2014 Sep 18;10(9):e1003836. doi: 10.1371/journal.pcbi.1003836 (PMC4168978; doi:10.1371/journal.pcbi.1003836)
Supplement: Figure S7 — Recombinational advantage declines with the density of local maxima. To clarify whether the time of maximal recombination advantage, , increases with decreasing density of local maxima, , we measured and in simulations, where is the mean density of maxima averaged over realizations as well as over genotype space (recall that the density of maxima is inhomogeneous in the RMF model). Panel (A) shows and panel (B) the corresponding maximal advantage vs. . Both quantities decline monotonically with increasing . The density was controlled by changing the slope for fixed (see inset of panel (A)). Data correspond to , and . (PDF) [file pcbi.1003836.s007.pdf]

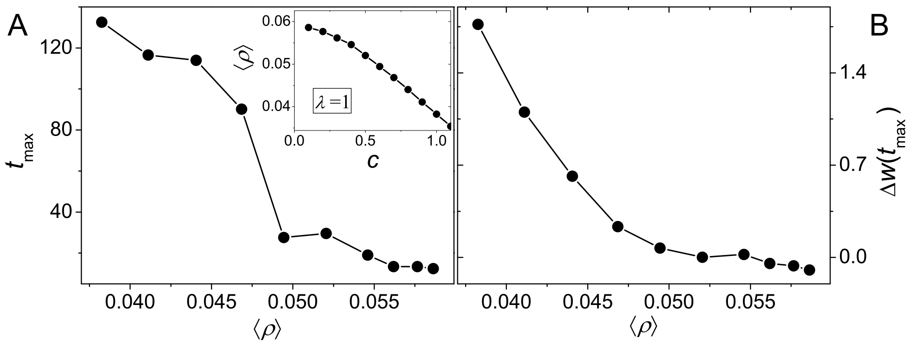

Figure S7. Recombinational advantage declines with the density of local maxima. To clarify whether the time of maximal recombination advantage, $t_{\max}$, increases with decreasing density of local maxima, ρ, we measured $\Delta w(t_{\max})(\langle\rho\rangle)$ and $t_{\max}(\langle\rho\rangle)$ in simulations, where $\langle\rho\rangle$ is the mean density of maxima averaged over realizations as well as over genotype space (recall that the density of maxima is inhomogeneous in the RMF model). Panel (A) shows $t_{\max}$ and panel (B) the corresponding maximal advantage $\Delta w(t_{\max})$ vs. $\langle\rho\rangle$. Both quantities decline monotonically with increasing $\langle\rho\rangle$. The density was controlled by changing the slope c for fixed $\lambda = 1$ (see inset of panel (A)). Data correspond to $N = 1000$, $N\mu = 4$ and $\lambda = 1$.
