# Supplementary material for: Multidimensional Epistasis and the Transitory Advantage of Sex
Source: PLoS Comput Biol. 2014 Sep 18;10(9):e1003836. doi: 10.1371/journal.pcbi.1003836 (PMC4168978; doi:10.1371/journal.pcbi.1003836)
Supplement: Figure S8 — (A), (C), and (E): Fitness velocities vs. for systems with parameters , , and . Intersections are marked by dashed lines. (B), (D), and (F): Corresponding curve for vs. . Compare with curves in the schematic pictures in fig. 8. (PDF) [file pcbi.1003836.s008.pdf]

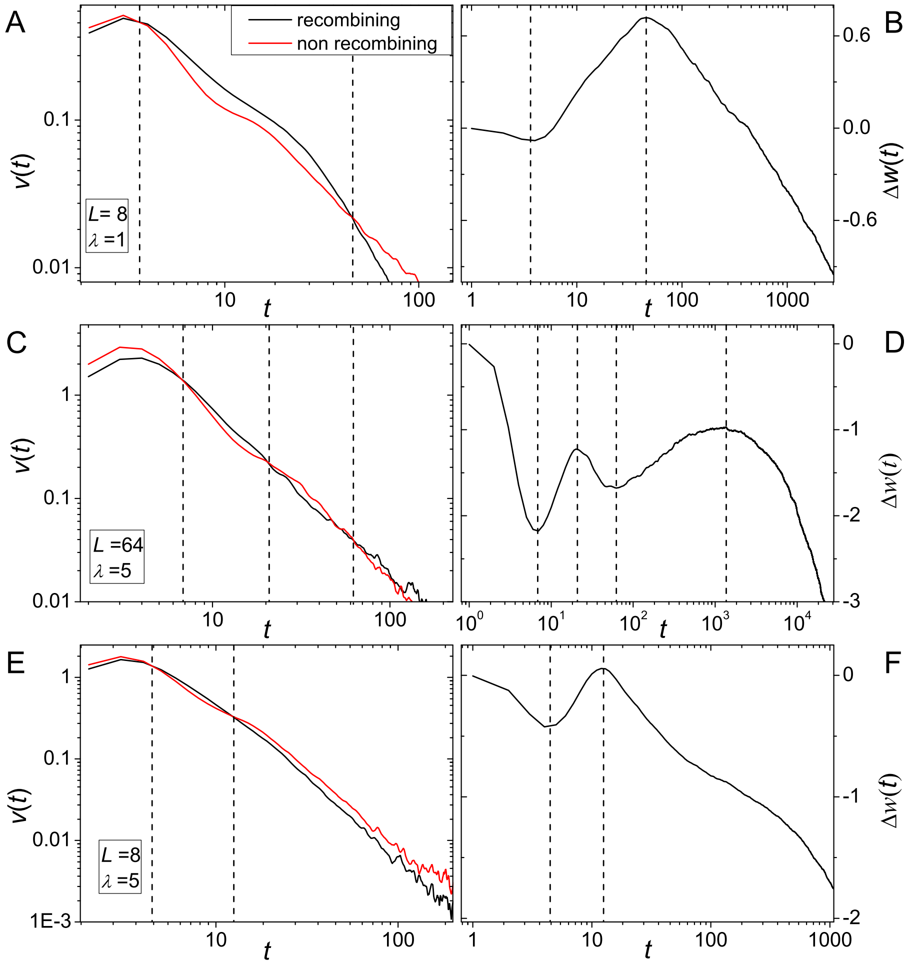


**Figure S8.** (A), (C), and (E): Fitness velocities vs. $t$ for systems with parameters $N = 2000$, $N\mu = 4$, and $c = 1$. Intersections are marked by dashed lines. (B), (D), and (F): Corresponding curve for $\Delta w$ vs. $t$. Compare with curves in the schematic pictures in fig. 8.
